# Dehydration‐driven organization of metabolites into NaDES‐like assemblies in orthodox seeds

**DOI:** 10.1111/nph.71080

**Published:** 2026-03-12

**Authors:** Youcef Haddad, Thomas Delhaye, Emmanuelle Limanton, Denis Morineau, Maëna Le Corvec, Christine Deponge, Anne Levrel, Alain Moréac, Virginie Nazabal, David Rondeau, Ludovic Paquin, Alain Bouchereau

**Affiliations:** ^1^ IGEPP UMR‐1349, INRAE University of Rennes, Institut Agro Rennes‐Angers Le Rheu F‐35650 France; ^2^ ISCR UMR‐6226 University of Rennes, CNRS, ENSCR Rennes F‐35042 France; ^3^ IETR UMR‐6164 University of Rennes Rennes F‐35042 France; ^4^ IPR UMR‐6251 University of Rennes, CNRS Rennes F‐35042 France; ^5^ ScanMAT University of Rennes, CNRS, ScanMAT‐UAR2025 Rennes F‐35042 France; ^6^ Metabolic Profiling and Metabolomic Platform (P2M2) METABOHUB, IGEPP Le Rheu F‐35000 France

**Keywords:** dehydration, desiccation tolerance, DESmolytes, molecular association, NaDES, oilseed rape (*Brassica napus*L.), seeds, vitrification

## Abstract

- Desiccation tolerance in plants, especially during orthodox seed dehydration, relies on compatible solute accumulation, complex molecular mechanisms, and intermolecular organizations that remain poorly understood.
- We combined metabolite profiling, mass spectrometry imaging, and electrospray and cold‐spray ionization mass spectrometry to investigate metabolite organization into natural deep eutectic solvent‐like assemblies during oilseed rape seed dehydration.
- We show that sucrose colocalizes and interacts with organic and amino acids in seed tissues to form hydration‐dependent, sucrose‐centered assemblies, whose formation is promoted by water loss and reproduced in bioinspired artificial mixtures displaying NaDES‐like physicochemical properties.
- These findings support the idea that seed metabolites, with sucrose as preferential matrix, organize into eutectic‐like supramolecular networks during dehydration, suggesting a physicochemical basis for cytoplasmic stabilization in desiccation‐tolerant seeds.

## Abstract 2

See also the Commentary on this article by Rolletschek & Borisjuk, **251**: 916–917.

## Introduction

Desiccation tolerance (DT), the remarkable capacity of anhydrobiotic organisms to survive extreme dehydration, is among the most striking examples of biological resilience across diverse taxa from plants and fungi to animals and microbes (Marks *et al*., 2025). DT is defined as the ability to withstand extreme water loss, reaching cellular water contents below 10% on a fresh weight (FW) basis, and to resume normal function upon rehydration without incurring irreversible damage (Leprince & Buitink, 2010; Dekkers *et al*., 2015; Roszkowska *et al*., 2023). Fundamentally, DT relies on complex biochemical and structural mechanisms that preserve cellular integrity during water loss and enable recovery upon rehydration (Walters *et al*., 2010; Wang *et al*., 2015; VanBuren *et al*., 2018). In orthodox seeds, desiccation, or late maturation, constitutes the final developmental phase following embryogenesis and maturation (Angelovici *et al*., 2010). During this stage, water content drops below *c*. 23% of FW, leading to a marked reduction in molecular mobility and ultimately, upon further dehydration, resulting in cytoplasmic vitrification and the formation of a glassy state below *c*.10% FW (Buitink & Leprince, 2008). Osmolytes capable of forming hydrogen‐bonding networks, especially nonreducing sugars such as sucrose, trehalose, and raffinose family oligosaccharides, play central roles in this process (Buitink & Leprince, 2004, 2008; Verdier *et al*., 2013; Matilla, 2022). These sugars form hydrogen‐bonded matrices that stabilize macromolecules and membranes while limiting oxidative damage during dehydration (Hoekstra *et al*., 2001; Berjak & Pammenter, 2013; Nicholson *et al*., 2025), particularly in the presence of divalent cations (Carpenter & Crowe, 1988; Billi & Potts, 2002). You *et al*. (2021) have shown that sugars can form stable hydrogen‐bond networks at lipid–water interfaces, helping preserve membrane structural integrity under dehydration stress. However, how these molecular interactions integrate into larger scale supramolecular organization *in vivo* remains to be fully resolved.

Over the past decade, hydrogen bond donor (HBD) and acceptor (HBA) molecules, such as sugars, amino acids, and organic acids, have gained attention as compatible components of natural deep eutectic solvents (NaDES) (Choi *et al*., 2011; Dai *et al*., 2013, 2015; Choi & Verpoorte, 2019; Bragagnolo *et al*., 2024). NaDES are defined as eutectic mixtures of natural compounds, typically composed of HBDs and HBAs, that at a specific molar ratio show a depressed melting temperature relative to their pure components, forming a stable liquid phase at or near room temperature (Smith *et al*., 2014; Martins *et al*., 2019). Synthetic NaDES have been the focus of a great deal of research, particularly for new applications such as biocatalysis (Lindberg *et al*., 2010; Khodaverdian *et al*., 2018; Buzatu *et al*., 2024) and green extraction of natural substances (Choi & Verpoorte, 2019; Gómez *et al*., 2019; Ivanović *et al*., 2020). Beyond their functional versatility, the natural occurrence of some NaDES constituents has prompted growing interest in their potential formation in living cells, from reactant metabolites accumulated especially under cellular stress conditions (Choi *et al*., 2011; du Toit *et al*., 2021; Durand *et al*., 2021; Farrant & Hilhorst, 2021; Vanda *et al*., 2021). The hypothesis that NaDES could form *in vivo* arose from three main observations. The first one is the high and often unexplained osmo‐induced concentration of some metabolites, considered as potential ingredients for NaDES in living cells (Choi *et al*., 2011; Dai *et al*., 2015, 2021). The second point is that these osmolytes, described as compatible solutes and kosmotropes, are thought to be involved in the thermodynamic stability of macromolecules since they are highly accumulated following stress, especially abiotic stress (Bubalo *et al*., 2023). Finally, plant exudates, saps, and nectar, which are very enriched in compounds that can exchange hydrogen bonds, such as sugars, organic acids, amino acids, and polyols, could have physical and thermodynamic aspects similar to NaDES (Durand *et al*., 2021; Vanda *et al*., 2021).

In desiccation‐tolerant seeds, all theoretical conditions for NaDES formation appear to be met, leading to the proposition that such mixtures may replace water and protect cellular structures during dehydration while maintaining their functionality (Choi *et al*., 2011; Durand *et al*., 2021). Dai *et al*. (2013) and du Toit *et al*. (2021) suggest that NaDES could act as mobility zones in the cytoplasm of dry tissues, whose role is to solubilize macromolecules and control rehydration. The establishment of high‐flux metabolic highways mediated by metabolon formation was proposed to be associated with the formation and predicted functionality of NaDES to explain the production and storage of natural products (Knudsen *et al*., 2020; Møller & Laursen, 2021). However, whether such assemblies retain liquid‐like properties or instead progressively lose molecular mobility and transition toward highly viscous or glassy states during dehydration remains unresolved. Importantly, these models remain largely conceptual, and direct experimental evidence for the *in vivo* formation, spatial organization, and functional properties of NaDES‐like assemblies in biological tissues is still lacking.

Here, we investigated the biochemical, metabolic, and physicochemical conditions conducive to the formation of NaDES‐like supramolecular assemblies in developing oilseed rape (*Brassica napus* L.) seeds. This annual crop of the Brassicaceae family is cultivated for its edible seed oil, protein meals, and biofuel use. Beyond their biochemical value, the physiological quality of seeds, defined by longevity and germination vigor, is a key target for breeders, which depends on a better understanding of the molecular determinants that underlie DT and survival in the dry state. Specifically, our first objective was to identify the osmolytes most likely to form NaDES in living seed cells, here termed ‘DESmolytes’ for the first time. We then examined their spatial colocalization within seed tissues, their capacity to interact *in vivo* and *in vitro*, and how dehydration reshapes these interactions and their physicochemical properties. Together, these objectives establish, for the first time, a mechanistic and experimental framework to test whether seed dehydration creates the physicochemical conditions required for NaDES formation *in vivo*.

## Materials and Methods

### Chemicals

Dipotassium malate was purchased from Cymit Quimica (Spain), and all other chemicals were purchased from Sigma‐Aldrich (France). Amino acid and organic acid standards were purchased in the L‐form, whereas monosaccharides were purchased in the D‐form. Coaxial inner tubes for Nuclear Magnetic Resonance (NMR) spectroscopy were obtained from Eurisotop (France).

### Seed collection

Developing seeds were harvested from oilseed rape (*Brassica napus* L. var. Aviso) plants grown in the field under conventional cropping conditions. Twenty plants were randomly selected from a homogeneous field plot. For each sampling date, 200 siliques (10 per plant) were collected, and the seeds extracted from all siliques were pooled to obtain a representative sample of the population at the corresponding developmental stage. Each pooled sample was divided into three biological replicates (*n* = 3) for analysis.

Six developmental stages were defined based on seed water content, which was estimated using the mean growing degree‐days accumulated from flowering (Bianchetti *et al*., 2021). Actual seed water content was determined from the difference between fresh and dry mass after immediate immersion in liquid nitrogen and freeze‐drying of representative seed samples collected at each developmental stage. Water content values represent means (*n* = 3). Seeds containing 75% (S1), 52% (S2), 39% (S3), 33% (S4), 20% (S5), and 5% (S6) water (expressed as % FW) were thus selected for analysis. Pooled seed samples were immediately frozen in liquid nitrogen and stored at −80°C until analyses.

### Liquid/liquid extraction for seed metabolite profiling

After freeze‐drying, representative seed samples from each developmental stage were ground in liquid nitrogen using a pestle and mortar. Approximately 10 mg of powder was then mixed with 1 ml of a MeOH/H_2_O/CHCl_3_ solution (4 : 4 : 2 v/v) containing β‐aminobutyric acid (baba) and adonitol for amino acid and carbohydrate internal standardization, respectively. After two rounds of agitation, for 15 and then 10 min, and a centrifugation step (5 min at 12 000 ***g***), the upper polar phase and the lower hydrophobic phase were separated (see extraction protocol in Methods S1 data).

Targeted analyses of amino acids on the one side, and sugars, organic acids, and polyols on the other side, were carried out from the polar fraction after the derivatization steps required before each analysis. Amino acid derivatization was carried out with 6‐aminoquinolyl‐N‐hydroxysuccinimidyl carbamate (AQC) (Waters AccQ‐Tag amino acid analysis system) as detailed in Methods S2 data. Amino acids were analyzed using an Acquity UPLC system (Waters Corporation, Milford, MA, USA) following the method described by Renault *et al*. (2010). One microliter of each derivatized extract was injected onto an Acquity UPLC BEH C18 column (1.7 μm, 2.1 × 100 mm; Waters) maintained at 55°C. The mobile phases consisted of AccQ‐Tag solution diluted 1 : 10 (v/v) (Eluent A; Waters) and pure acetonitrile (Eluent B). The flow rate was set to 0.7 ml min^−1^, and the following gradient program was applied: initial, 99.9% A; 2 min, 99.9% A; 6 min, 98% A; 10 min, 96% A; 12 min, 80% A; 15 min, 40.4% A; 16 min, 40.4% A; 17 min, 99.9% A; and 18 min, 99.9% A. Amino acids were detected at 260 nm using a photodiode array detector.

For the simultaneous analysis of sugars, organic acids, and polyols, trimethylsilylation was performed on the dry extract residue following Gravot *et al*. (2010). The dried extract was resuspended in 50 μl of methoxyamine/pyridine solution (20 mg ml^−1^) and incubated for 90 min at 30°C in a dry heating block. Then, 50 μl of N‐methyl‐N‐(trimethylsilyl)trifluoroacetamide (≥ 95% purity) was added, and the mixture was incubated for 30 min at 37°C (Methods S3). Adonitol, included at the beginning of extraction, was used as the internal standard.

Derivatized extracts were injected into a gas chromatograph equipped with a flame ionization detector (GC‐FID; Trace 1300; Thermo Fisher Scientific, Waltham, CA, USA), fitted with a TriPlus RSH autosampler, a split/splitless injector (split ratio 1 : 25) set at 260°C, and a TG‐5MS column (30 m × 0.32 mm × 0.25 μm; Thermo Fisher Scientific). The FID temperature was set to 310°C. The oven temperature program was as follows: 4 min at 70°C, ramped at 10°C min^−1^ to 198°C, held for 2 min, then 1°C min^−1^ to 202°C, 15°C min^−1^ to 268°C, held for 3 min, followed by 1°C min^−1^ to 272°C, and finally 10°C min^−1^ to 310°C, held for 7 min (Gravot *et al*., 2010).

All extraction, derivatization, and quantification procedures were performed on three independent biological replicates (*n* = 3).

### Mass spectrometry analysis

Different mass spectrometry techniques were employed to analyze both seed samples and artificial solutions. Methanol/water (50 : 50, v/v) extracts from S3 seeds (39%; expressed as % FW) were first examined by direct infusion mass spectrometry (DIMS) using an electrospray ionization (ESI) source with the expectation of catching specific noncovalent bonding adducts (Yamaguchi, 2013). The choice of S3 seeds at 39% water content was made to ensure that the DT phase was fully established, based on Li *et al*. (2011), who reported that *B. napus* seeds acquire complete tolerance below 50% water content. Because metabolite concentrations varied among seed extracts, artificial solutions of methanol/water (50 : 50, v/v) or pure acetonitrile containing equimolar concentrations of putative NaDES constituents (DESmolytes) were also analyzed. The artificial solutions were composed of sucrose, dipotassium l‐malate, tripotassium citrate, gamma‐aminobutyric acid (Gaba), l‐asparagine, l‐glutamine, potassium l‐aspartate, and potassium l‐glutamate standards. Binary solutions for all possible combinations of these compounds were also injected. All detected adducts were fragmented at 10 eV collision energy to confirm the presence of noncovalent interactions. Analyses were carried out on a Bruker timsTOF Pro 2 HRMS equipped with an ESI source operating in negative mode under the following conditions: capillary voltage, 4.5 kV; nebulizer gas (N_2_), 2.2 bar; dry gas flow, 10 l min^−1^; dry heater, 220°C; ion mobility parameters: mode custom from 0.45 to 1.4 1/K_0_; ramp time, 100 ms; accumulation time, 100 ms; duty cycle, 100%.

To capture highly labile interactions potentially disrupted under ESI conditions, a standard solution containing sucrose, dipotassium l‐malate, and tripotassium citrate was also analyzed using cold‐spray ionization mass spectrometry (CSI‐MS). This variant of the ESI source, in which the metal capillary used for the infusion of the analyte solution can be lowered until −40°C, maintains the noncovalent complex assemblies or favors the aggregation of analyzed molecules during the spray formation (Yamaguchi, 2003, 2013). As a result, CSI‐MS has already been used for the characterization of DES by mass spectrometry (Percevault *et al*., 2021; Bertrand *et al*., 2025). CSI‐MS analyses were performed using the AccuTOF CS instrument (JEOL, Tokyo, Japan). The needle voltage was set to −1.5 kV, with nitrogen as nebulizer and dry gas at flow rates of 1.0 and 2.0 l min^−1^, respectively. The nebulizer gas temperature ranged from −40°C to 0°C, and the desolvation chamber was maintained below 30°C. The ring lens and ion guide voltages were set to −15 and − 29 V, respectively. Further details on the CSI technique are provided in Percevault *et al*. (2021). ESI and CSI analyses were performed in triplicate (*n* = 3).

To assess the spatial colocalization of putative DESmolytes within seed tissues, mass spectrometry imaging (MSI) was performed using desorption electrospray ionization (DESI) at 100 μm spatial resolution. Representative seed samples from each developmental stage were embedded in carboxymethyl cellulose using Peel‐Away disposable molds, cured for 2 h on dry ice, and stored at −80°C for at least 12 h. Sections (60 μm thick) were cut at −20°C using a Leica CM3050 S cryostat (MX35 Ultra low‐profile blades, 34° angle) and mounted on SuperFrost™ glass slides. Sections from the six developmental stages (75, 52, 39, 33, 20, and 5% water content, expressed as % FW) were mounted on the same slide and analyzed simultaneously. The slide, containing a minimum of five seeds per developmental stage (Fig. S1), was analyzed once. For each developmental stage, the entire surface of each seed section was scanned by DESI‐MSI. For quantitative analyses, regions of interest corresponding to specific tissues were selected as square areas of 100 μm × 100 μm, and six independent pixel regions were extracted per tissue and per seed section (Fig. S2).

DESI‐MSI analyses were performed on a Xevo G2‐XS QTof HRMS instrument (Waters, Milford, MA, USA) equipped with a DESI 2D stage operating in negative mode with the following parameters: spray voltage, 2 kV; solvent, methanol/water (98 : 2, 0.1% formic acid); flow rate, ~5 μl min^−1^; nebulizing gas, nitrogen at *c*. 5 bar; sprayer angle, 75°; X‐Y step size, 100 μm. Mass spectra were acquired with a 5‐s scan time, source temperature of 120°C, and sampling cone voltage of 40 V. Data were processed using the HDI software (Waters Corporation). Targeted imaging of DESmolytes and their binary adducts was performed by extracting the 5000 most intense ions within the *m/z* 100–1200 range to visualize putative *in situ* molecular interactions.

### Statistical analyses

All statistical analyses were performed using RStudio (v.4.3.1). For metabolite profiling, concentrations were log_10_‐transformed before statistical testing. The effect of developmental stage and biological replicate was assessed using a linear model. Planned pairwise comparisons of each developmental stage relative to stage S6 were then performed using Welch's *t*‐tests, which do not assume equal variances and are robust for small sample sizes. *P*‐values were adjusted for multiple testing using the Benjamini–Hochberg false discovery rate correction. For graphical representation, mean metabolite concentrations were calculated from nontransformed data, whereas statistical significance was derived from analyses performed on log‐transformed values.

For quantitative DESI analyses, malate and adduct ion intensities were extracted on a pixel‐by‐pixel basis from DESI‐MS images. The adduct proportion was calculated as adduct ion intensity / (malate ion intensity + adduct ion intensity). A linear mixed‐effects model was used to test the global effect of developmental stage on relative adduct proportion, with developmental stage included as a fixed effect and compartment as a random effect. In addition, nonparametric Kruskal–Wallis tests followed by pairwise Wilcoxon tests with Holm correction were performed separately for each compartment to compare developmental stages (S2, S5, and S6).

### NaDES preparation

Based on DESmolytes identified in seeds through metabolite profiling and imaging, some ternary NaDES were prepared by mixing two solid synthetic compounds with water. For all mixtures, water content is expressed as a percentage of the total mass (w/w). Considering seed dehydration, water was initially added at more than 50% (w/w) before eliminating a fraction using desiccator or vacuum concentrator (SpeedVac). The use of heating, often carried out for NaDES preparation, has not been prioritized in order to maintain dehydration conditions as close as possible to physiological conditions. Water was then added if necessary to bring the content up to the target level. Water content was measured with the Karl Fisher titration method (Gallina *et al*., 2010) using a Metrohm 795 KFT Titrino apparatus. Sucrose, mixed at equimolar ratio (1/1, mol/mol) with dipotassium l‐malate, tripotassium citrate, γ‐aminobutyric acid, l‐asparagine, l‐glutamine, or potassium l‐glutamate, was involved in all the mixtures prepared. All NaDES were prepared in triplicate (*n* = 3).

### Differential scanning calorimetry (DSC) analyses

The phase transitions of two prepared NaDES, namely sucrose/dipotassium l‐malate and sucrose/potassium L‐glutamate (1/1; mol/mol), were determined by Differential Scanning Calorimetry (DSC) using a Q‐20 TA Instrument equipped with a liquid nitrogen cooling system. The melting transition of an indium sample was used for calibration of temperature and heat flux. The samples were sealed in Tzero© aluminum pans. NaDES were analyzed with the following water contents: 15, 20, 30, 40, 50, 60, and 70% (w/w). The temperature ranged from −130°C to 20°C with a heat flow of 10°C min^−1^. Sucrose, malate, and glutamate have also been analyzed separately in aqueous solutions at 50% and 40% water contents (w/w). DSC analyses were performed in triplicate (*n* = 3).

### Nuclear Magnetic Resonance (NMR) spectroscopy

For hydrogen bonding characterization, a sucrose/dipotassium l‐malate (1/1, mol/mol) mixture was prepared with water content ranging from 20% to 70% (w/w) in 10% intervals. Each water content reference corresponds to a separately prepared sample, rather than a single mixture that was stepwise dehydrated. This approach ensures consistent solute ratios and controlled hydration levels for each measurement. Those mixtures were analyzed through ^1^H and selective 1D Nuclear Overhauser Effect SpectroscopY (NOESY) NMR. Diffusion‐Ordered SpectroscopY (DOSY) was also carried out to determine the number of diffusion coefficients in each mixture. Analyses were performed at 298 K using Bruker AV III HD 500 MHz spectrometer fitted with a double resonance broad band (BBFO model) probe. To avoid contact between samples and the reference solvent, NaDES mixtures were transferred to 4‐mm coaxial inner cells. The cells were then placed in conventional NMR tubes containing CD_3_OD.

### Mid‐infrared (MIR) spectroscopy

To detect specific signals of hydrogen bond formation, the NaDES solutions were analyzed using mid‐infrared (MIR) spectroscopy. Similar to the NMR analyses, each sucrose/dipotassium l‐malate mixture for MIR spectroscopy was independently prepared at defined water contents of <10, 15, 20, 30, 40, 50, 60, and 70% (w/w). MIR spectra were recorded with a Spectrum 3‐PerkinElmer Fourier transform infrared spectrometer equipped with a diamond attenuated total reflection (ATR) accessory. Spectra were acquired in the 3800–600 cm^−1^ range, with 32 scans, a resolution of 4 cm^−1^, and an interferogram velocity of 0.5 cm^−1^. Two microliters of each sample were deposited onto the diamond ATR, and spectra were collected every 2 min until signal stabilization was achieved in order to assess the influence of water desorption on the spectra.

To optimize signal resolution, spectra were processed in R using the Savitzky–Golay method, applying second derivatives with a 23‐point window in the 3700–2800 and 1800–600 cm^−1^ regions. The positions of spectral peaks, full widths at half maximum, and band intensities were compared. Additionally, the hydroxyl group (OH) band (2800–3700 cm^−1^) was deconvoluted using PeakFit software, employing eight Voigt profile functions. The position and area of the bands were also compared. NMR and MIR analyses were performed in triplicate (*n* = 3).

## Results

### Identification of putative DESmolytes in oilseed rape seeds

To identify putative NaDES‐forming metabolites, we first profiled and quantified simple sugars, organic acids, polyols, and amino acids in developing *B. napus* seeds. Table 1 summarizes metabolite contents expressed in μmol g^−1^ dry weight (DW) (see Table S1, for SD values). A linear model including developmental stage and biological replicate revealed a significant effect of developmental stage on metabolite concentrations, whereas no significant effect of biological replicate was detected (linear model, *P* < 0.05 for developmental stage; *P* > 0.05 for biological replicate). Planned pairwise comparisons relative to the final maturation stage (S6; 5% FW), performed on log‐transformed data, revealed significant differences for several metabolites. Overall, most metabolites were more concentrated in the early, highly hydrated seeds and progressively declined during seed filling and desiccation.

**Table 1:** Concentration of major sugars, polyols, amino acids, and organic acids in *Brassica napus* L. seeds across six developmental stages.

	S1	S2	S3	S4	S5	S6
Sucrose	67.47	51.55	62.36	64.64	70.64	57.77
Malate	19.39***	16.58**	17.05***	7.00**	5.90**	3.75
Citrate	25.87**	8.81**	7.04*	3.99*	3.58	1.96
Glucose	14.41*	1.90**	0.82*	0.43	0.31	0.13
Fructose	13.21*	1.30*	0.72*	0.09	0.04	0.01
Myo‐inositol	1.95*	0.87**	0.56**	0.34	0.36	0.20
Galactose	0.37	0.22	0.03	0.48	0.07	0.02
Galactinol	0.00	0.05	0.40	0.42	0.43	0.35
Raffinose	0.00*	0.61*	0.44	0.28	0.25	0.21
Succinate	2.22*	0.37*	0.32*	0.35*	0.12	0.07
Glycerate	1.48*	0.18***	0.04	0.05	0.03	0.01
Fumarate	0.90	0.25	0.18	0.16	0.13	0.04
Histidine	1.09	0.00	0.00	0.00	0.00	1.81
Asparagine	1.81	1.07	2.85	4.29	5.31	2.85
Serine	0.76	1.16*	0.88*	0.34	0.56	0.21
Glutamine	33.18*	2.91*	1.59*	0.35	0.26	0.13
Glycine	1.38*	0.31	0.21	0.35	0.28	0.07
Aspartate	0.75	1.12	2.27	1.49	2.84	1.86
Glutamate	10.90	2.68	3.21	5.16	5.38	4.04
Threonine	2.45***	0.52	0.54	0.22	0.23	0.20
Alpha‐Alanine	6.37**	1.40*	1.40	1.18**	0.88	0.51
Gaba	11.77*	4.70**	2.87***	3.15**	2.73**	0.15
Proline	3.31***	1.22*	0.63	0.36	0.52	0.32
Lysine	1.31*	0.34	0.24	0.13	0.13	0.23
Tyrosine	1.27*	0.36	0.22	0.09	0.11	0.21
Valine	5.64**	0.82*	0.74	0.57	0.66	0.48
Isoleucine	2.45**	0.28	0.21	0.15	0.00	0.00
Leucine	1.58	1.41	0.35	0.29	0.45	0.80
Phenylalanine	2.01*	0.93	0.44	0.37*	0.29	0.24

Concentrations are expressed in μmol g^−1^ dry weight (DW). Developmental stages were defined according to seed water content (% fresh weight, FW): 75% (S1), 52% (S2), 39% (S3), 33% (S4), 20% (S5), and 5% (S6). Values represent means of three biological replicates (*n* = 3); corresponding SD are provided in Table S1. Color intensity indicates mean metabolite concentrations, ranging from “under the limit of quantification” (0.00 μmol g^−1^ DW; blue) to 70 μmol g^−1^ DW (magenta), with intermediate shades indicating intermediate concentrations. Asterisks indicate statistically significant differences relative to stage S6 based on Welch's *t*‐test with Benjamini–Hochberg false discovery rate correction (*, *P* < 0.05; **, *P* < 0.01; ***, *P* < 0.001).

Based on both their hydrogen‐bonding potential (as donors or acceptors) and their relative abundance during intermediate maturation stages (S2–S5; 52–20% FW), when DT is supposed to be acquired, sucrose, malate, citrate, asparagine, glutamate, γ‐aminobutyrate (Gaba), glutamine, and aspartate were selected as DESmolyte candidates (Fig. S3). Across all developmental stages, sucrose was by far the most abundant polar metabolite and did not exhibit significant variation in content across seed water contents. Glutamate, aspartate, and asparagine exhibited similar developmental trends, though at lower absolute levels. Organic acids were considered in their likely physiological salt forms, with potassium as the predominant counterion. Since l‐amino acids and d‐configured sugars are the predominant biologically relevant forms in plant tissues, only these stereoisomers were considered in this study.

### *In vitro* affinity between putative seed NaDES forming ingredients

**Fig. 1:**
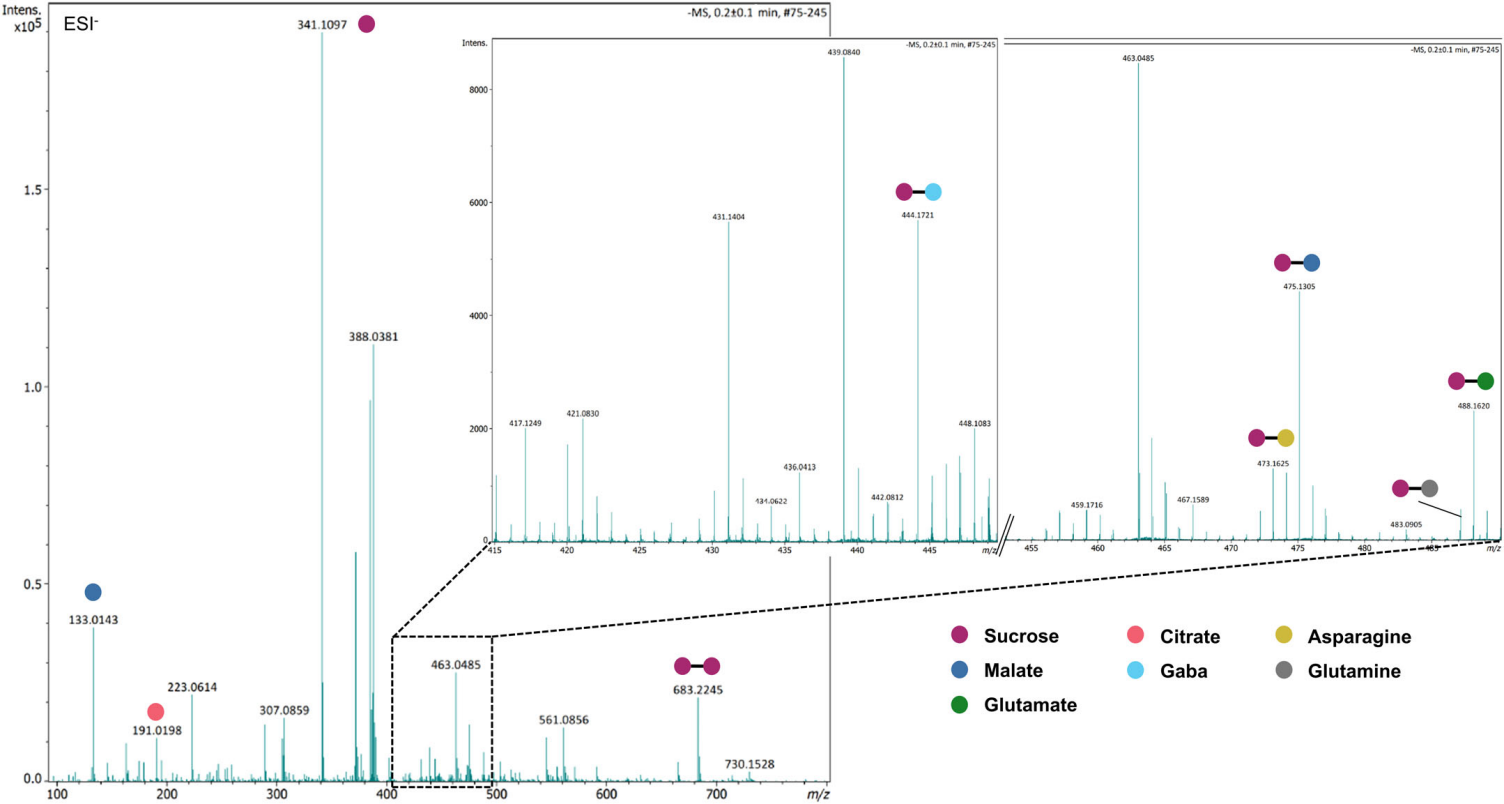
Detected DESmolytes and their corresponding heterodimers in a polar extract of *Brassica napus* L. seeds at the S3 developmental stage (39% water content, % fresh weight). The extract was analyzed by direct infusion in negative‐mode electrospray ionization. Detected DESmolyte ions are annotated with single‐colored dots, while dimeric adducts detected between *m/z* 415 and 490 are indicated by pairs of linked dots, each color representing one constituent monomer.

The capacity of the selected DESmolytes (sucrose, malate, citrate, asparagine, glutamate, γ‐aminobutyrate (Gaba), glutamine, and aspartate) to form noncovalent molecular associations (electrostatic or hydrogen bonds) was assessed in both seed polar extracts and solutions of pure molecules. Except for glutamine, all compounds were detected individually in the polar extract of S3 seeds (39% FW) using ESI. Remarkably, sucrose consistently formed preferential adducts with all of these metabolites except citrate, both in complex seed extracts and in reconstructed binary mixtures (Fig. 1). No adducts were observed between any other metabolite pairs in the absence of sucrose.

In both natural and artificial mixtures, six sucrose‐based adducts were identified with a mass accuracy < 5 ppm: *m/z* 444.1704; *m/z* 473.1604; *m/z* 474.1468; *m/z* 475.1293; *m/z* 487.1751; and *m/z* 488.1604 (Table S2). These correspond to dimers involving sucrose and, respectively, Gaba, asparagine, aspartate, malate, glutamine, and glutamate. Each dimer involved a single sucrose partner interaction, and fragmentation below 10 eV confirmed their noncovalent nature. Replacing sucrose with trehalose or raffinose, which are regularly found in nature under water deficit, produced similar adducts. In contrast to sucrose, trehalose, and raffinose, the reducing monosaccharides glucose and fructose did not form any detectable adducts under the same experimental conditions, despite being present at comparable concentrations in the artificial solutions (Fig. S4). Citrate did not form any detectable adducts even in binary solutions.

**Fig. 2:**
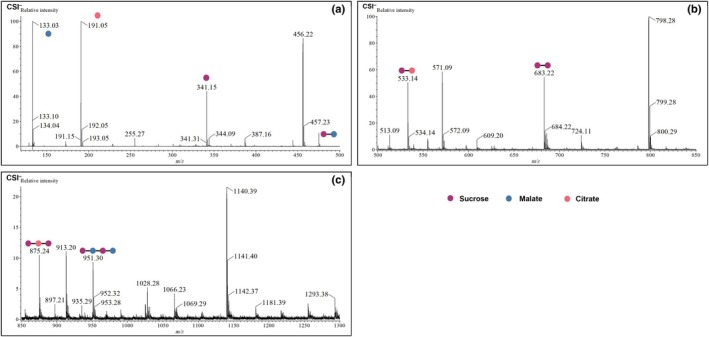
Mass spectra of an equimolar aqueous solution containing sucrose, malate, and citrate. (a), (b), and (c) correspond to the *m/z* ranges 100–500, 500–850, and 850–1300, respectively. The solution was analyzed at −20°C by direct infusion using negative‐mode cold‐spray ionization. As in electrospray ionization, DESmolyte ions are indicated by single‐colored dots, whereas dimeric and oligomeric adducts are represented by two or more linked dots, each color denoting one constituent monomer.

CSI analysis further refined these observations (Fig. 2). All detected assemblies were sucrose‐centered adducts. Although the deprotonated ions of malate (*m/z* 133), citrate (*m/z* 191), and sucrose (*m/z* 341) were individually observed, no assemblies involving malate or citrate in the absence of sucrose were detected. Interestingly, CSI revealed additional sucrose‐citrate adducts and higher order oligomers containing multiple sucrose units, which were absent under ESI. Notably, the malate–malate association observed under CSI was detected only within larger sucrose‐containing assemblies.

### *In vivo* localization and interaction between seed DESmolytes

**Fig. 3:**
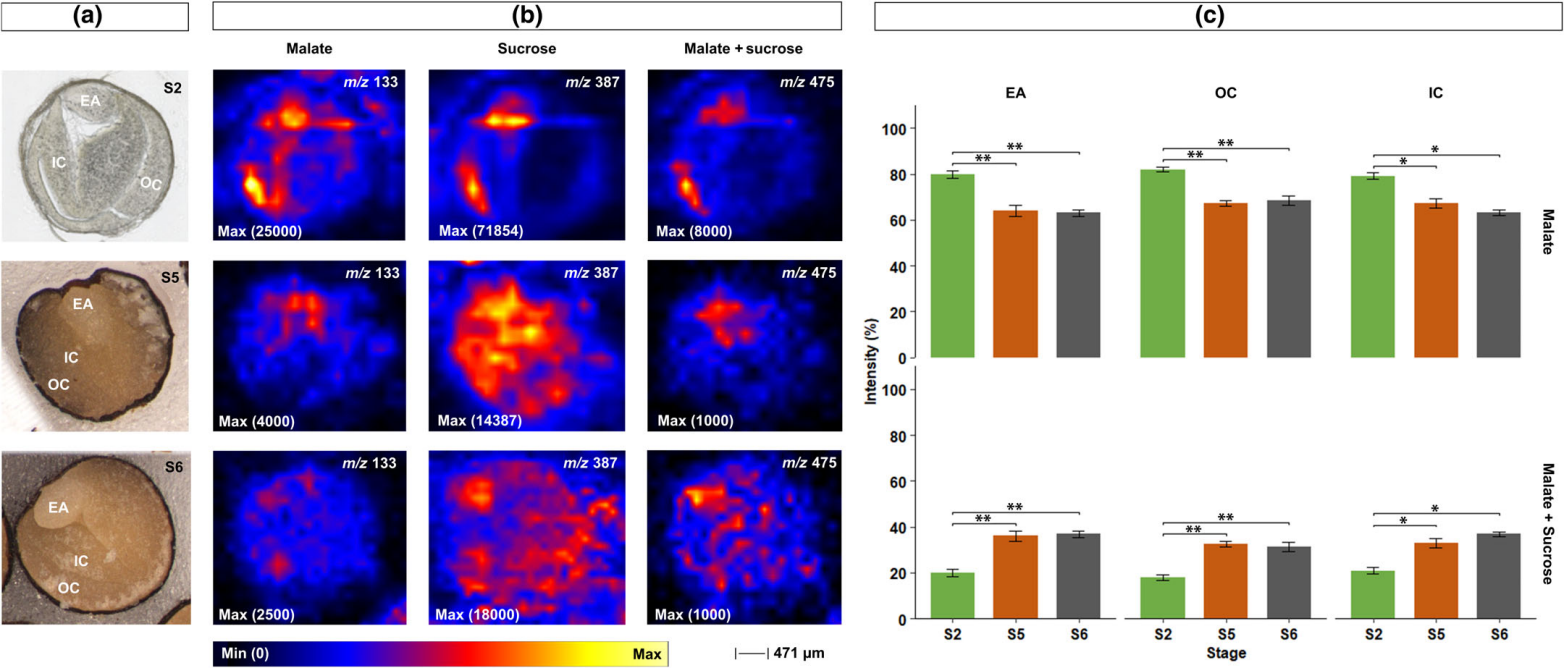
Spatial distribution of malate (*m/z* 133), sucrose (*m/z* 387), and their heterodimer (*m/z* 475) in *Brassica napus* L. seeds at three developmental stages defined by seed water content (% fresh weight): 52% (S2), 20% (S5), and 5% (S6). (a) Microscopic images showing the main seed compartments: embryonic axis, inner cotyledons, and outer cotyledons. (b) Desorption electrospray ionization (DESI) images illustrating the spatial distribution of malate, sucrose, and the sucrose‐malate heterodimer. Signal intensity ranges from minimum (black, 0) to maximum (yellow), with the maximum intensity for each molecule indicated in parentheses. DESI images were acquired at 100‐μm spatial resolution. For each biological replicate, the entire seed surface was analyzed, providing full coverage of all tissue regions at each developmental stage. (c) Relative intensities of malate (*m*/*z* 133) and sucrose‐malate adduct (*m*/*z* 475) in representative pixels from each compartment and developmental stage. Values are expressed as percentages of the summed intensities (*m*/*z* 133 + *m*/*z* 475) and shown as means ± SD (*n* = 6). Statistical significance between developmental stages was assessed using Kruskal–Wallis tests followed by pairwise Wilcoxon tests with Holm correction (*, *P* < 0.05; **, *P* < 0.01).

The native spatial distribution of identified DESmolytes and adducts was examined by MSI. Microscopic images of *B. napus* seed sections at three developmental stages (S2, S5, and S6) corresponding to 75, 20, and 5% water content (% FW), respectively, are shown in Fig. 3(a). The main seed compartments, namely the embryonic axis (EA), inner cotyledons (IC), and outer cotyledons (OC), were clearly distinguishable.

At the S2 early stage, both malate (*m/z* 133) and sucrose (*m/z* 387) were predominantly detected in the EA and the IC, and their heterodimer (*m/z* 475) was also observed in these regions (Fig. 3b). At S5, sucrose was broadly distributed across all seed compartments, whereas malate and the corresponding sucrose adduct remained mainly localized in the embryonic axis. In the driest stage (S6), malate intensity dropped sharply in the EA, while sucrose and the adduct persisted throughout all compartments. Overall, the sucrose‐malate adduct displayed the same distribution as its partners, with variable relative intensities across developmental stages, and the EA appeared as the preferential site of heterodimer formation.

Semi‐quantitative analysis of ion intensities from representative pixel mass spectra across seed compartments and developmental stages revealed marked stage‐dependent changes in malate and sucrose‐malate heterodimer relative abundances. A linear mixed‐effects model (developmental stage as a fixed effect and compartment as a random effect) showed a significant effect of developmental stage on relative adduct proportion (*F* = 82.5, *P* < 0.0001), with increases of +14.2% at S5 and + 15.4% at S6 relative to S2. At S2, malate accounted for *c*. 80% of the combined malate + sucrose‐malate signal, whereas the heterodimer contributed *c*. 20%. As seeds matured and dehydrated, malate signal significantly declined, reaching *c*. 60% at S6, while the sucrose‐malate signal increased to *c*. 40% at S6 (Fig. 3c). These stage‐dependent shifts in relative ion proportions were consistently observed across compartments (Wilcoxon tests with Holm correction, *P* < 0.01 and *P* < 0.05). Other DESmolytes, including glutamate (*m/z* 146), aspartate (*m/z* 132), and asparagine (*m/z* 131), and their sucrose‐based adducts (*m/z* 488, *m/z* 474, and *m/z* 473, respectively) exhibited comparable spatial dynamics (Fig. S5). These patterns were reproducible across biological replicates.

### Phase transitions of seed bioinspired‐NaDES

Thermal transitions of sucrose‐malate and sucrose‐glutamate artificial NaDES (1 : 1; mol/mol) were determined using DSC at water contents ranging from 15 to 50% (w/w). For comparison, individual aqueous solutions of sucrose, malate, and glutamate were also analyzed at 50% water content (w/w).

**Fig. 4:**
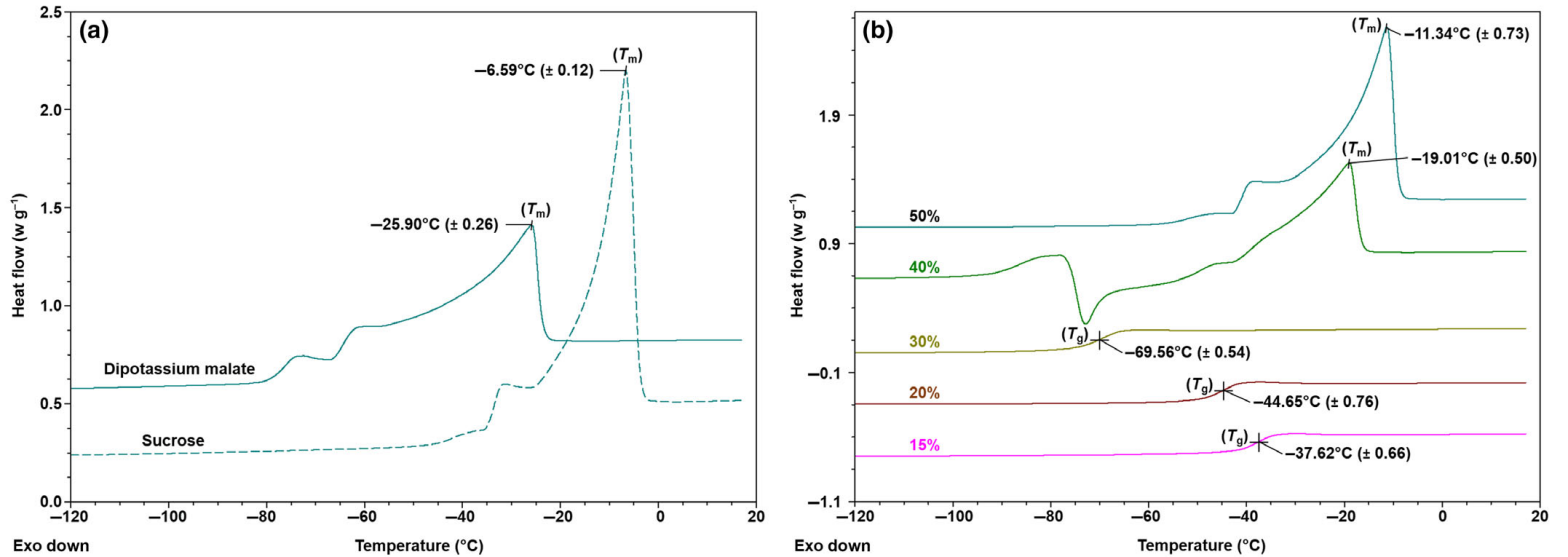
Phase transitions of individual solutions and natural deep eutectic solventmixtures of sucrose/dipotassium malate (1/1; mol/mol) between 20 and −120°C. (a) Thermograms showing the melting points (*T*_m_) of individual aqueous solutions of sucrose and malate (1 g ml^−1^). (b) Thermograms showing the glass transitions (*T*_g_) and melting points (*T*_m_) of the mixture between 15 and 50% water content (w/w). Values represent means ± SD (*n* = 3).

Aqueous sucrose and dipotassium malate solutions showed melting onsets at −36°C and −60°C with peak maxima *c*. −6°C and −25°C, respectively (Fig. 4a). In NaDES mixtures, endothermic melting signals were also observed at −11°C (50% water) and −19°C (40% water) (Fig. 4b). The 50% water mixture also exhibited crystallization upon cooling (−34°C), while at 40% water, crystallization occurred only during heating (−73°C), indicating an improved stability of the supercooled liquid with respect to crystallization on decreasing the hydration level (Fig. S6).

At water contents ≤ 30% by mass (w/w), no exothermic or endothermic signals were observed. Instead, NaDES exhibited a single glass transition (*T*_g_), whose temperature increased as water content decreased. Recalling that the calorimetric *T*_g_ is classically related to the temperature at which the liquid relaxation time approaches the value *τ* ≈ 10^2^ s, the observed variation of *T*_g_ reflects the increase of viscosity of NaDES on decreasing water content. The sucrose‐glutamate NaDES displayed comparable phase transition behavior across the same range of water contents. Notably, aqueous sucrose solutions also exhibited glass transitions at 30% and 20% water (w/w); however, these transitions were accompanied by crystallization and melting signals (Fig. S7).

### H‐bonding characterization in reconstituted NaDES from seed composition

**Fig. 5:**
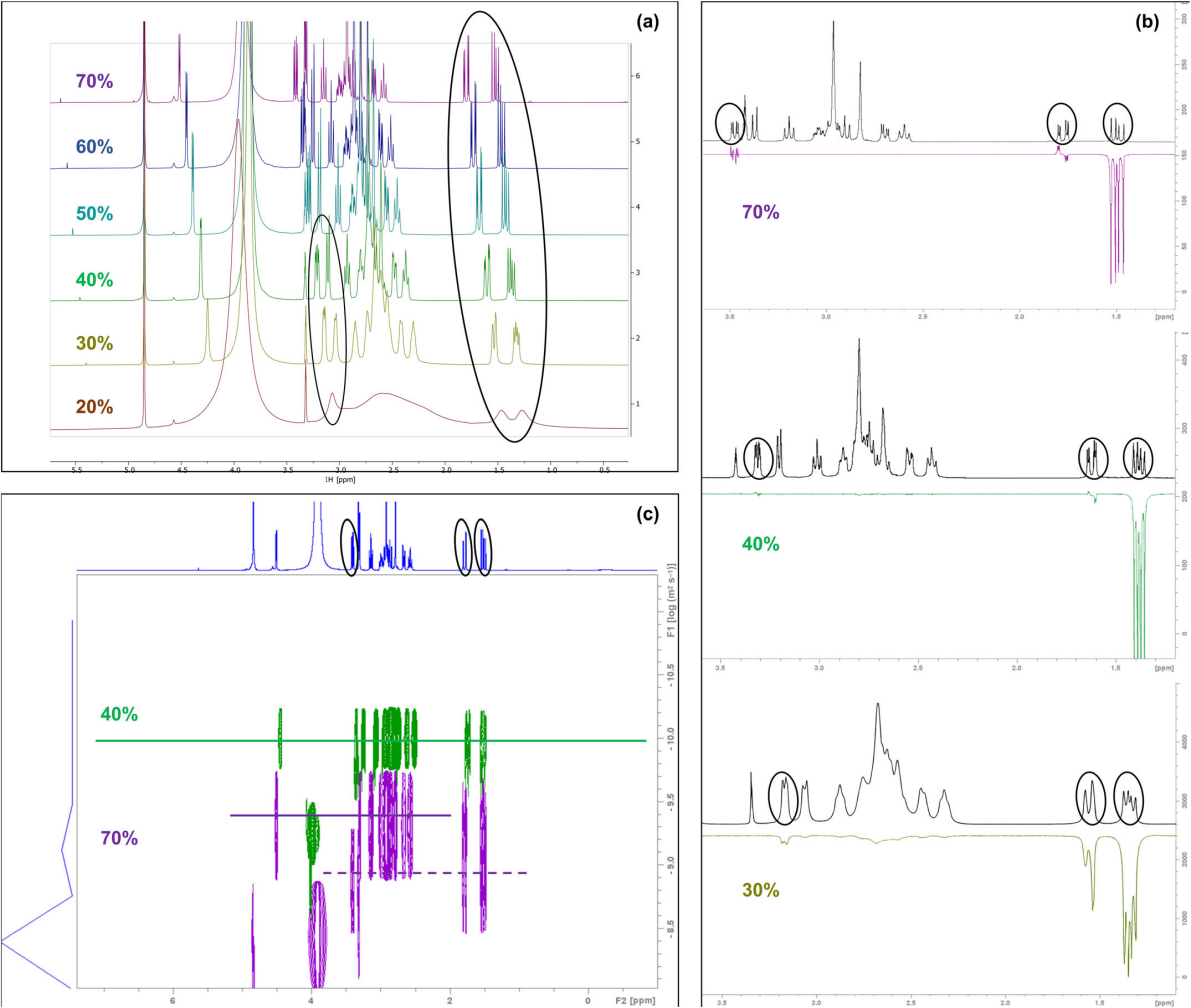
500 MHz liquid‐state NMR spectra of a sucrose/dipotassium malate mixture (1:1, mol/mol). Malate signals are indicated with black circles. (a) Evolution of one‐dimensional ^1^H spectra as a function of water content, ranging from 20% to 70% (w/w). (b) Selective one‐dimensional Nuclear Overhauser Effect SpectroscopY spectra recorded at 30, 40, and 70% water content. Reference ^1^H spectra for each hydration level are presented in black, while spectra obtained after irradiation of a malate signal are presented in purple (70%), green (40%), and olive green (30%). Malate signals are again circled in black. (c) Diffusion‐Ordered SpectroscopY spectra of natural deep eutectic solvent solutions at 70% (purple) and 40% (green) water content. Diffusion coefficients are indicated by lines colored according to the water content of the solution.

A sucrose/dipotassium malate (1/1; mol/mol) mixture containing 20–70% water (w/w) was used to characterize hydrogen bonding between molecular partners. The superimposition of the ^1^H‐NMR spectra showed a progressive upfield shift (up to 0.3 ppm) as water content increased, indicating enhanced molecular mobility (Fig. 5a). Spectral sharpening confirmed improved resolution at higher hydration levels.

Selective 1D NOESY experiments showed that irradiation of a malate resonance (*c*. 1.5 ppm) led to only intramalate correlations (with 1.75 and 3.4 ppm resonances) at 70% water (Fig. 5b). At 40% water, additional sucrose‐malate correlations (NOEs) appeared. These sucrose‐malate NOEs became stronger and more distinct at 30% water, consistent with tighter intermolecular contacts upon dehydration. The presence of such supramolecular complexes was further supported by DOSY analysis (Fig. 5c). At 70% water, diffusion coefficients of 123.4 μm^2^ s^−1^ (malate), 87.0 μm^2^ s^**−**1^ (sucrose), and 224.0 μm^2^ s^**−**1^ (water) were distinct, whereas at 40% water, sucrose and malate shared a slower diffusion rate (45.4 μm^2^ s^**−**1^), indicating molecular association and reduced mobility.

**Fig. 6:**
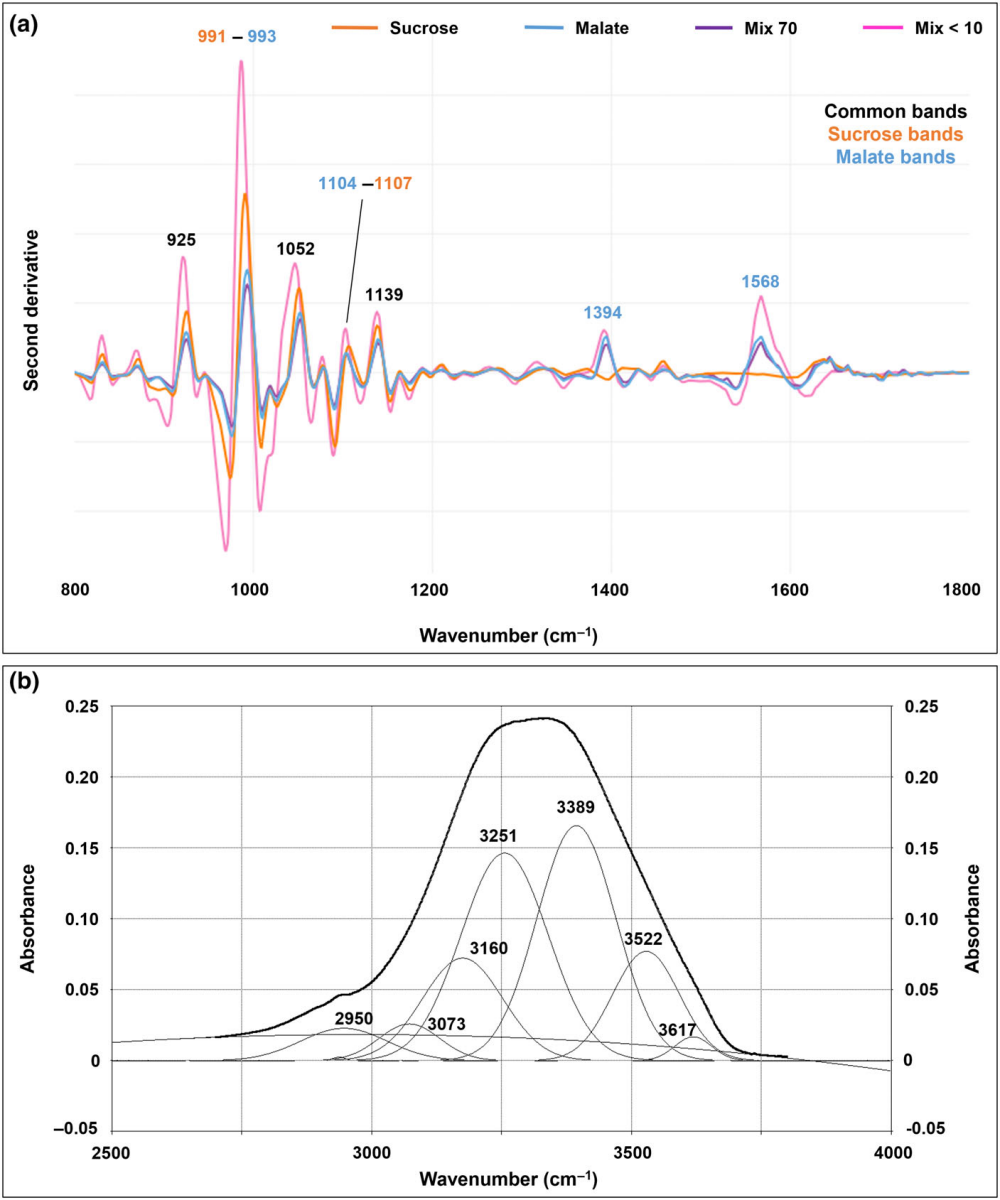
Mid‐infrared spectra of pure sucrose and dipotassium malate compounds as well as their aqueous mixture solutions at 70% (Mix 70; w/w) and less than 10% (Mix < 10; w/w) water content. (a) Second derivative in the 800–1800 cm^−1^ region for malate (blue), sucrose (orange), Mix 70 (purple), and Mix < 10 (pink). Common bands are indicated in black while sucrose and malate bands are indicated with their respective colors. (b) Decomposition of the Mix 70 signal between 2700 and 3700 cm^−1^ spectral region.

Complementary insights were obtained by MIR. Individual sucrose and malate solutions displayed characteristic absorbance bands between 800 and 1600 cm^−1^ (925, 1052, 1139, 991–993, 1104–1107, 1394, 1568 cm^−1^; Fig. 6a). In the 2800–3700 cm^−1^ region, the C‐H stretching zone (2830–3010 cm^−1^) and the broad O‐H stretching band (3000–3700 cm^−1^) dominated (Fig. 6b). Deconvolution using Voigt profile fitting revealed consistent maxima with both sucrose (3619, 3524, 3390, 3259, 3175, 3078, and 2922 cm^−1^) and malate (3617, 3523, 3392, 3256, 3166, 3077, and 2940 cm^−1^) vibrational modes. In the mixtures, spectra were dominated by malate features (1104, 922 cm^−1^) and exhibited marked intensity changes with decreasing water content: increases at 989, 922, 2940 cm^−1^, and decreases at 1395, 1139, 1104, 1052 cm^−1^. High‐frequency bands near 3517 and 3620 cm^−1^, associated with free water, diminished strongly as hydration declined.

A decrease in water content in the mixtures also induced red‐shifts (shifts toward lower frequencies) of several bands compared with pure malate. In the 800–1600 cm^−1^ region, these shifts ranged from 7 cm^−1^ at 993 cm^−1^ (δC‐H/C‐C) to 6 cm^−1^ at 1052 cm^−1^ (ν(C‐O)/C‐C) and 4 cm^−1^ at 925 cm^−1^ (δ(C‐H)). Shift amplitudes decreased to *c*. 2 cm^−1^ at 1394 cm^−1^ (ν_s_(COO^−^)) and became negligible (≤ 1 cm^−1^) at 1567 cm^−1^ (νₐ_s_(COO^−^)), 1139 cm^−1^, and 1104 cm^−1^ (ν(C‐O)) (Table 2). More pronounced red‐shifts (up to 30 cm^−1^ at 2910 cm^−1^) were observed in the O‐H stretching (Table 2). These shifts were particularly significant in NaDES containing ≤ 30% water.

**Table 2:** Positions (cm^−1^) of main dipotassium malate and sucrose bands obtained by mid infrared analyses.

	Malate	Sucrose	Mix 70	Mix 60	Mix 50	Mix 40	Mix 30	Mix 20	Mix 15	Mix < 10
OH‐band decomposition	3617	3619	0	−1	1	−3	−10	−17	−18	na
	3523	3524	−1	3	0	−1	−3	−5	−2	−6
	3392	3390	−3	2	−2	−1	0	0	3	−4
	3256	3259	−5	0	3	−2	−1	−4	−2	−6
	3166	3175	−6	6	9	−9	−17	−17	−14	−20
	3077	3078	−4	−1	0	−7	−14	−13	−14	−13
	2940	2922	10	−4	−17	−11	−19	−27	−35	−30
*ν_as*(COO^−^)	1567	NA	0	0	0	0	−1	0	0	0
*ν_s*(COO^−^)	1394	NA	1	0	1	−1	−1	−2	−2	−2
>*ν*(C‐O)	1139	1139	0	0	−1	0	0	0	−1	−1
*ν*(C‐O)	1104	1107	0	0	2	−1	−1	−1	−1	−1
*ν*(C‐O/ C‐C)	1052	1051	0	0	−1	−1	−3	−4	−4	−6
*δ*(C‐H/C‐C)	993	991	1	0	−2	−2	−3	−5	−5	−7
*δ*(C‐H)	925	935	0	0	0	−1	−2	−3	−3	−4

Equimolar sucrose‐malate mixtures (Mix) with varying water contents exhibited shifts in band positions relative to pure malate. Shift values are reported as means (*n* = 3) and expressed in cm^−1^. Water content is expressed as % H_2_O (w/w) relative to the total mass of sucrose + malate. Mixture solutions are denoted as ‘Mix’ followed by their water content (e.g. Mix 70 = 70% H_2_O (w/w); Mix < 10 = < 10% H_2_O (w/w)). Analyses were carried out at 22°C.

Drying MIR experiments confirmed that these spectral changes reflected reorganization of hydrogen‐bonding networks rather than simple concentration effects. During water evaporation, intensities of the 1800–800 cm^−1^ bands increased, accompanied by red‐shifts in samples with ≥ 40% H_2_O (Table 3). Below 30% water, band positions stabilized, indicating minimal free water. Sucrose exhibited stronger red‐shifts than malate, suggesting a more extensive hydrogen‐bonding network. Technical replicates confirmed the high reproducibility of band positions in the 1800–800 cm^−1^ region, processed using second‐derivative analysis, including drying‐related shifts (≤ 2 cm^−1^). In the 2800–3700 cm^−1^ region, C‐H and O‐H stretching bands were analyzed by peak fitting, with replicates performed for 30% and 60% H_2_O. These replicates confirmed the trends observed in Tables 2 and 3; for example, the band at *c*. 2920 cm^−1^ shifted by 22–32 cm^−1^ at 60% H_2_O compared with 7–8 cm^−1^ at 30% H_2_O during drying, with differences in band position reflecting the precision of the decomposition procedure.

**Table 3:** MIR band shifts, relative to initial positions (IP), measured for sucrose/dipotassium malate mixtures at varying water contents, as well as for their respective individual aqueous solutions, after drying on an attenuated total reflectance (ATR) crystal.

	IP (cm^−1^)	Malate	Sucrose	Mix 70	Mix 60	Mix 40	Mix 30	Mix 20	Mix 15	Mix < 10
OH‐band decomposition	3617	6	20	24	2	9	2	0	0	0
	3523	5	14	13	0	8	2	0	0	0
	3392	15	11	6	3	0	0	0	0	0
	3256	14	13	0	9	5	0	0	0	0
	3166	19	43	18	38	15	4	0	0	0
	3077	14	26	13	21	10	2	0	0	0
	2940	20	7	29	15	6	3	0	0	0
*ν_as*(COO^−^)	1567	0	na	0	0	0	−1	0	0	0
*ν_s*(COO^−^)	1394	2	na	2	1	1	1	0	0	0
*ν*(C‐O)	1139	0	2	0	0	0	0	1	0	0
*ν*(C‐O)	1104	1	2	1	1	0	0	0	0	0
*ν*(C‐O/ C‐C)	1052	3	4	3	3	2	0	0	0	0
*δ*(C‐H/C‐C)	993	4	5	5	4	2	1	0	0	0
*δ*(C‐H)	925	2	12	2	2	1	0	0	0	0

Water content of mixtures is expressed as % H_2_O (w/w) relative to total solute mass (e.g. Mix 70 = 70% H_2_O; Mix < 10 = < 10% H_2_O). Individual solutions were analyzed at 1 g ml^−1^. Analyses were carried out at 22°C.

Collectively, NMR and MIR analyses reveal a hydration‐dependent transition from weakly associated solutes to a stable, structured hydrogen‐bond network typical of NaDES organization.

## Discussion

The ability of orthodox seeds to survive extreme dehydration has long been associated with the accumulation of nonreducing sugars and compatible solutes capable of establishing hydrogen‐bonding networks (Hoekstra *et al*., 2001; Verdier *et al*., 2013; He *et al*., 2024). These compounds are thought to stabilize cellular structures and macromolecules during the transition from a hydrated cytoplasm to a glassy state. However, the precise molecular organization underlying this protective mechanism has remained unclear. Our combined *in vivo* and *in vitro* analyses provide converging evidence that mixtures of seed metabolites find favorable cellular physicochemical conditions to form NaDES‐like supramolecular assemblies during seed maturation and dehydration.

The *in vivo* metabolite profiling exploration revealed that the first prerequisite for NaDES formation, namely the coexistence of compatible hydrogen‐bond donor and acceptor molecules in water‐limited cell environments, for which the name DESmolytes is proposed here for the first time, is satisfied in developing *B. napus* seeds (Table 1). The high accumulation of sucrose, together with detectable levels of free organic and amino acids such as malate, citrate, glutamate, and aspartate, provides a reliable chemical perimeter for NaDES formation. Sucrose was by far the most abundant solute, suggesting that it could act as a central DESmolyte capable of interacting with multiple partners. The persistence of these metabolites up to advanced dehydration stages (20–5% FW) supports their potential involvement as versatile constituents of spatially localized intracellular eutectic phases.

Direct infusion mass spectrometric evidence confirmed that these solutes interact via noncovalent bonds. Sucrose consistently formed adducts with several amino and organic acids, while no other metabolite pairs displayed similar associations (Figs 1, 2). This selectivity likely results from the exceptionally high concentration of sucrose and its chemical affinity with diverse molecules, which increases the probability of forming heterodimers and higher order supramolecular assemblies. In this context, di‐ and trisaccharides appear as central scaffolds for hydrogen‐bonded molecular networks, explaining their recurrent implication in DT across taxa. Notably, trehalose and raffinose exhibited comparable associative behavior, while glucose and fructose did not, reinforcing the key role of nonreducing sugars. Within seed tissues, in which molecular mobility is severely limited, the colocalization of interacting DESmolytes in ≤100 μm regions makes their interaction inevitable during drying at the intratissular/intracellular levels. In dry seeds, the increasing intensity of the sucrose‐malate dimer, as free malate decreases, confirms that this interaction is promoted by desiccation (Fig. 3). This implies that DESmolytes become immobilized in time and space in specific conformations, enabling the formation of stable and localized supramolecular complexes. This scenario is supported by ion mobility mass spectrometry data, showing that each dimer exhibits a unique drift time, implying a single stable conformation *in vitro* (Fig. S8). DESI‐MSI further indicates that sucrose‐malate adducts are broadly distributed across seed compartments, with the notable exception of the seed coat, and display a pronounced occurrence in the embryonic axis. However, a more refined assessment of quantitative differences between compartments would require higher‐resolution approaches.

The physicochemical behaviors of artificial NaDES mixtures, mimicking the observed *in vivo* compositions under controlled dehydration conditions, provide mechanistic insight into these associations. DSC revealed strong cryoscopic depression (decrease) of the ice melting point, consistent with strongly bound water and the decrease of its activity in the presence of NaDES‐forming solutes (Fig. 4). The magnitude of melting‐point depression increased with decreasing hydration, demonstrating that these systems effectively stabilize water in a supercooled or glassy state. Crystallization was progressively suppressed below 40% water, and only glass transitions were detected at ≤ 30% water, indicating vitrification of these systems. This behavior is consistent with cytoplasmic vitrification in seeds, which becomes fully established at lower water contents, *c*. 10% FW (Buitink & Leprince, 2004, 2008). This suggests that these NaDES have the necessary physical properties to integrate into the cellular environment and undergo vitrification once free water has been slowly eliminated.

Hydrogen‐bond characterization through NMR and MIR spectroscopy further revealed progressive, hydration‐dependent organization of the sucrose/dipotassium malate mixture. NMR data indicated that dehydration promotes tighter intermolecular contacts and the formation of supramolecular assemblies at ≤ 30% water (Fig. 5). Complementary MIR analyses supported these conclusions, as the sucrose/dipotassium malate mixture exhibited changes in band intensities and pronounced red‐shifts below 30% water (Fig. 6; Table 2), reflecting progressive reorganization of hydrogen‐bonding networks. Notably, the magnitude of the red‐shifts was not uniform across all vibrational bands, suggesting structural heterogeneity in the hydrogen‐bond network, with some functional groups participating in stronger or more numerous hydrogen bonds than others. In other words, different molecular moieties respond differently to dehydration, consistent with the formation of a complex, spatially heterogeneous NaDES‐like supramolecular network rather than a uniform matrix. MIR drying experiments confirmed that these spectral changes were not due to simple concentration effects: Below 30% water, band positions stabilized, indicating minimal free water and formation of a highly structured network (Table 3). This network likely integrates residual water into stable intermolecular interactions, forming a highly viscous, amorphous matrix under extreme dehydration conditions, consistent with observations in seed tissues below *c*. 23% FW (Buitink & Leprince, 2008). While this reconstituted network mimics conditions observed in seed cytoplasm, the exact *in vivo* behavior of NaDES‐like assemblies under extreme dehydration remains to be directly verified.

It is important to note that most of our *in vitro* prepared NaDES exhibited similar properties, and the sucrose‐dipotassium malate combination was chosen for illustration purposes. The choice of malate salt instead of its acidic form is justified by the near‐neutral cytoplasmic pH, while potassium was selected as the most relevant counter‐ion, being the predominant inorganic cation in seeds (Rolletschek *et al*., 2021). In this context, the incidence and role of potassium in the formation of supramolecular structures will need to be studied.

Together, these findings provide, for the first time, direct experimental support that NaDES‐forming metabolites can colocalize and interact within living seed tissues, fulfilling the key physicochemical prerequisites for natural deep eutectic phase formation *in vivo*. Among the metabolite combinations tested, sucrose‐malate, sucrose‐glutamate, and sucrose‐citrate emerge as the most promising NaDES candidates in *B. napus* seeds, whereas other combinations could be considered in other plant tissues exposed to dehydration. The close correspondence between *in vivo* and *in vitro* behaviors, particularly the hydration‐dependent reorganization of sucrose‐centered molecular assemblies and the inhibition of crystallization with decreasing water content, demonstrates that dehydration drives both systems toward comparable supramolecular states. This convergence (Table 4) supports the notion that, as cellular water content declines, sucrose‐based assemblies with diverse favorable molecular partners reorganize into stable supramolecular networks resembling NaDES. Importantly, in fully dry seeds, these assemblies likely integrate into a glassy, quasi‐solid cytoplasmic state, suggesting that fluid NaDES‐like zones are absent. This scenario remains plausible under conditions in which water is relatively homogeneously distributed throughout the tissue, although the presence of highly localized regions with elevated water content cannot be excluded and could transiently allow more liquid‐like behavior. During intermediate dehydration stages (*c*. 20–50% water content), NaDES‐like assemblies may retain more liquid‐like properties, potentially enabling functional macromolecular interactions. In *B. napus*, DT is reported to initiate at relatively high water contents and to become fully established *c*. 50% water content (Li *et al*., 2011), a range that partially overlaps with hydration levels at which eutectic or NaDES‐like behaviors may become physically plausible. That said, the formation of stable NaDES is generally considered unlikely at higher water contents, in which excess water disrupts eutectic organization, and it therefore remains to be demonstrated whether similar assemblies can form during drying in seeds that are still desiccation‐sensitive. Consequently, any potential protective or functional roles of NaDES‐like assemblies, either during intermediate dehydration stages or in fully dry seed tissues, must be directly tested in future experiments.

**Table 4:** Comparative evidence of *in vivo* (*Brassica napus* L. seeds) and *in vitro* (artificial NaDES mixtures) observations.

Observation	*In vivo* (seeds)	*In vitro* (artificial NaDES mixtures)	Convergence
Spatial organization	Colocalization of DESmolytes within seed compartment (≤ 100 μm regions; DESI)	Spatial proximity between DESmolytes under limited water conditions (NOESY, DOSY)	Restricted molecular mobility enhances proximity and favors molecular association
Molecular interactions	Sucrose‐based heterodimers detected by MSI and DIMS	Noncovalent dimers confirmed by ESI/CSI with sucrose‐centered complexes	Sucrose acts as central H‐bond donor/acceptor hub
Hydrogen‐bond network	Sucrose‐malate heterodimer intensity increases during dehydration (DESI)	Stabilization of hydrogen‐bonding network at < 30% water (MIR)	Dehydration promotes and stabilizes supramolecular assemblies
Hydration‐dependent physical stat	Cytoplasmic vitrification below *c*. 10% FW (Buitink & Leprince, 2004, 2008)	No NaDES or ice crystallization is observed. Glass transition (*T*_g_) detected at ≤ 30% water content (DSC)	Comparable hydration range for glass formation
Water activity	Low mobility and bound‐state water in mature seed	Strong depression (decrease) of ice melting and reduced water activity (DSC)	Decrease in water freedom in both systems

By linking *in vivo* metabolite organization with *in vitro* NaDES behavior, this study provides a framework for understanding the physicochemical principles underlying eutectic microdomain formation in desiccation‐tolerant organisms. Techniques such as MIR spectroscopy and high resolution magic angle spinning (HR‐MAS) NMR were explored on seed tissues, but broad absorption bands and dominant lipid signals limited the detection of specific metabolite interactions. Improving sample preparation could help overcome these limitations. Complementary approaches such as atomic force microscopy (AFM), Raman imaging, micro‐rheology, or HR‐MAS NMR under controlled hydration could enable more direct comparisons between biological tissues and artificial NaDES systems. Future work should also evaluate biological activities of these eutectic mixtures for possible DT features such as interactions with macromolecules under dehydration or heat stress, potential solubilization of metabolites, and extend these observations to other seeds and desiccation‐tolerant systems such as pollen, lichens, and resurrection plants.

## Competing interests

None declared.

## Author contributions

YH developed the study design, proposed additional experiments and refined the research approach during the course of the work, performed the experiments, analyzed the data, interpreted the data and wrote the initial draft of the manuscript. TD proposed additional experiments, performed experiments, analyzed the data, participated in data interpretation, reviewed and edited the manuscript. EL performed experiments, analyzed the data, reviewed and edited the manuscript. DM analyzed the data, participated in data interpretation, reviewed and edited the manuscript. MLC performed experiments, analyzed the data, participated in data interpretation, reviewed and edited the manuscript. CD performed experiments, analyzed the data, participated in data interpretation, reviewed and edited the manuscript. AL performed experiments, analyzed the data, reviewed and edited the manuscript. AM proposed additional experiments, analyzed the data, participated in data interpretation, reviewed the manuscript. VN proposed additional experiments, reviewed the manuscript. DR proposed additional experiments, performed experiments, analyzed the data, participated in data interpretation, reviewed and edited the manuscript. LP and AB designed the project, developed the study design, proposed additional experiments, and refined the research approach during the course of the work, participated in data interpretation, reviewed and edited the manuscript, and acquired funding.

## Disclaimer

The New Phytologist Foundation remains neutral with regard to jurisdictional claims in maps and in any institutional affiliations.

## Supporting information


**Fig. S1** Microscopic and DESI images representing the spatial distribution of malate (*m*/*z* 132), sucrose (*m*/*z* 387), and glutamate (*m*/*z* 146), as well as heterodimers formed between malate and sucrose (*m*/*z* 475), and glutamate and sucrose (*m*/*z* 488) in *Brassica napus* L. seeds at six different developmental stages.
**Fig. S2** Representative DESI pixels (left) from each *Brassica napus* L. seed compartment at three developmental stages defined by seed water content (% fresh weight): 52% (S2), 20% (S5), and 5% (S6).
**Fig. S3** Contents of selected DESmolytes, expressed in μmol g^−1^ of dry weight (μmol g^−1^ DW), in *Brassica napus* L. seeds across six developmental stages.
**Fig. S4** Mass spectra representing a comparative analysis of three standard solutions containing DESmolytes at equimolar concentration.
**Fig. S5** Spatial distribution of additional DESmolytes and their heterodimers in dry *Brassica napus* L. seeds.
**Fig. S6** Phase transitions of sucrose/dipotassium malate mixtures at different water contents.
**Fig. S7** Phase transitions of sucrose/water solution at different water contents.
**Fig. S8** Ion mobility of detected heterodimers in *Brassica napus* L. seed extract at the S3 developmental stage.
**Methods S1** Detailed protocol for amino acids, sugars, organics acids, and polyols extraction.
**Methods S2** AccQTag derivatization for amino acid analysis.
**Methods S3** Trimethylsilylation for GC‐FID analysis.
**Table S1** Standard deviations associated with the contents of major sugars, polyols, amino acids, and organic acids in *Brassica napus* L. seeds across six developmental stages.
**Table S2** Detected heterodimers in an artificial solution containing putative DESmolytes identified in *Brassica napus* L. seeds.
Please note: Wiley is not responsible for the content or functionality of any Supporting Information supplied by the authors. Any queries (other than missing material) should be directed to the *New Phytologist* Central Office.

## Data Availability

Detailed experimental protocols, instrumental parameters, and data processing procedures are described in the Materials and Methods section and in the Supporting Information (Methods S1–S3; Figs S1, S2). All data generated during this study are available either in the article or in the Supporting Information section. Quantitative metabolite profiling data across developmental stages are provided in Tables 1 and S1. Mass spectrometry data, including electrospray and cold‐spray ionization adduct assignments and desorption electrospray ionization mass spectrometry imaging data, are presented in Figs 1, 2, 3 and Supporting Information Data (Table S2; Figs S4, S5). Physicochemical datasets obtained by differential scanning calorimetry, nuclear magnetic resonance spectroscopy, and mid‐infrared spectroscopy are provided in Figs 4, 5, 6 and Tables 2, 3, as well as in Figs S6 and S7.
