# Supplementary material for: Dehydration‐driven organization of metabolites into NaDES‐like assemblies in orthodox seeds
Source: New Phytol. 2026 Mar 12;251(3):1172–87. doi: 10.1111/nph.71080 (PMC13326504; doi:10.1111/nph.71080)
Supplement: Supplementary file 1 — Fig. S1 Microscopic and DESI images representing the spatial distribution of malate (m/z 132), sucrose (m/z 387), and glutamate (m/z 146), as well as heterodimers formed between malate and sucrose (m/z 475), and glutamate and sucrose (m/z 488) in Brassica napus L. seeds at six different developmental stages. Fig. S2 Representative DESI pixels (left) from each Brassica napus L. seed compartment at three developmental stages defined by seed water content (% fresh weight): 52% (S2), 20% (S5), and 5% (S6). Fig. S3 Contents of selected DESmolytes, expressed in μmol g−1 of dry weight (μmol g−1 DW), in Brassica napus L. seeds across six developmental stages. Fig. S4 Mass spectra representing a comparative analysis of three standard solutions containing DESmolytes at equimolar concentration. Fig. S5 Spatial distribution of additional DESmolytes and their heterodimers in dry Brassica napus L. seeds. Fig. S6 Phase transitions of sucrose/dipotassium malate mixtures at different water contents. Fig. S7 Phase transitions of sucrose/water solution at different water contents. Fig. S8 Ion mobility of detected heterodimers in Brassica napus L. seed extract at the S3 developmental stage. Methods S1 Detailed protocol for amino acids, sugars, organics acids, and polyols extraction. Methods S2 AccQTag derivatization for amino acid analysis. Methods S3 Trimethylsilylation for GC‐FID analysis. Table S1 Standard deviations associated with the contents of major sugars, polyols, amino acids, and organic acids in Brassica napus L. seeds across six developmental stages. Table S2 Detected heterodimers in an artificial solution containing putative DESmolytes identified in Brassica napus L. seeds. Please note: Wiley is not responsible for the content or functionality of any Supporting Information supplied by the authors. Any queries (other than missing material) should be directed to the New Phytologist Central Office. [file NPH-251-1172-s001.pdf]

New phytologist Supporting Information

Article title: Dehydration-driven organization of metabolites into NaDES-like assemblies in orthodox seeds

Authors: Youcef Haddad, Thomas Delhaye, Emmanuelle Limanton, Denis Morineau, Maëna Le Corvec, Christine Deponge, Anne Levrel, Alain Moréac, Virginie Nazabal, David Rondeau, Ludovic Paquin, Alain Bouchereau

Article acceptance date: 15 February 2026

Methods S1. Detailed protocol for amino acids, sugars, organics acids, and polyols extraction

- Weigh 9-11 mg of freeze-dried tissue powder into Eppendorf tubes.
- Add 500 µL of methanolic BABA/adonitol solution to each tube.
- Vortex.
- Shake for 15 min.
- Add 250 µL of chloroform to each tube. Vortex.
- Shake for 10 min.
- Add 500 µL ultrapure water.
- Vortex for 20 s.
- Centrifuge for 5 min at 12,000 × g at 15°C.
- Transfer the upper polar phase to a 1.5 mL Eppendorf tube.
- Aliquot 50µL of each sample into Eppendorf tubes and evaporate using a Speedvac for 1 h 30 min to 2 h at 35°C.

Methods S2. AccQTag derivatization for amino acid analysis

- Use an AccQTag derivatization kit (Waters)
- Turn on a water bath at 55°C (1 h heating time required)
- Dissolve the derivatization reagent in 1 mL acetonitrile (heat for 2 min only).
- For each sample, in an Eppendorf tube, Add 35 µL borate buffer, 5 µL of polar extract, and 10 µL AccQ-Tag derivatization reagent
- Vortex.
- Briefly centrifuge the tubes.
- Incubate for 10 min at 55°C in the water bath.
- Transfer the contents to polypropylene UPLC vials with integrated insert reducers.

- Prepare an external standard (ES) following the same protocol, replacing the seed extract with a solution containing all amino acids at identical concentrations.

Methods S3. Trimethylsilylation for GC-FID analysis

For 18 samples:

- Weigh 20 mg of methoxyamine into an Eppendorf tube.
- Add 1 mL pyridine.
- Vortex briefly.
- Place on a shaker at 800 rpm for 15 min.
- Vortex for an additional 1 min.
- Add 50 µL of the solution to each tube containing the dried extracts and external standards.
- Incubate for 90 min at 30°C in a dry bath.
- Add 50 µL MSTFA per tube (stored at 4°C).
- Incubate for 30 min at 37°C in a dry bath.
- Keep samples at room temperature for at least 4 h before GC injection.

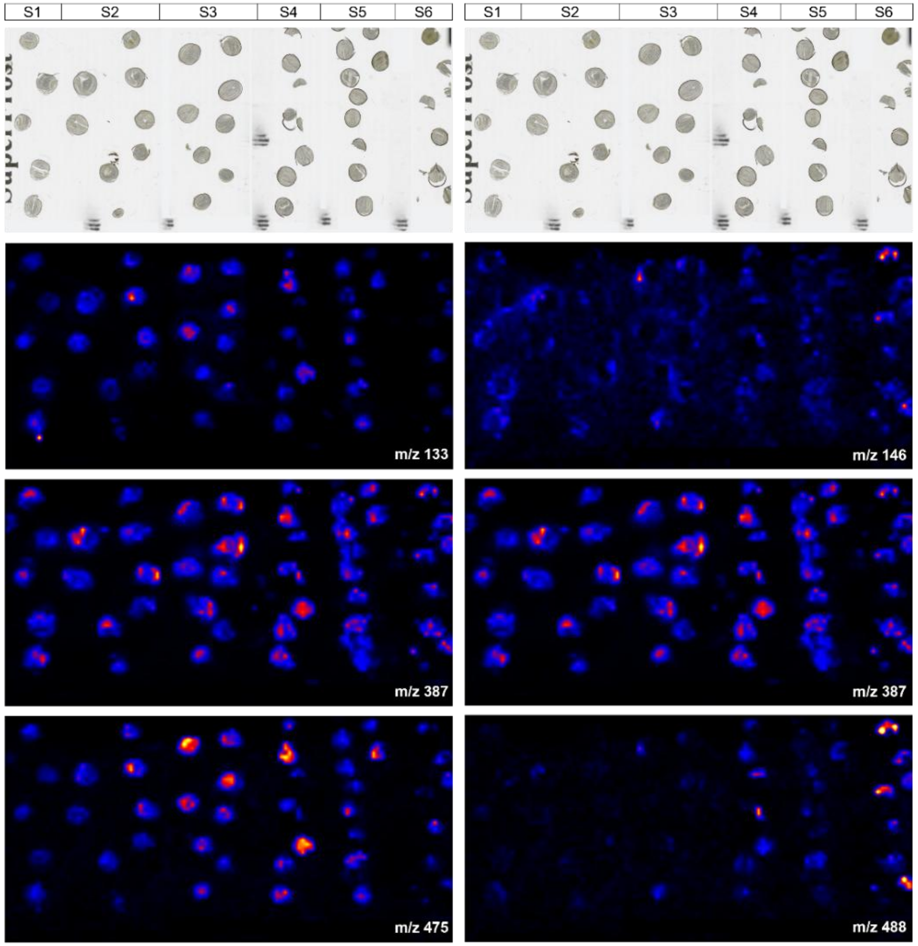

Fig. S1. Microscopic and DESI images representing the spatial distribution of malate (m/z 132), sucrose (m/z 387), and glutamate (m/z 146), as well as heterodimers formed between malate and sucrose (m/z 475), and glutamate and sucrose (m/z 488) in *Brassica napus* L. seeds at six different developmental stages. Developmental stages were defined based on seed water content expressed as a percentage of fresh weight (% FW): 75% (S1), 52% (S2), 39% (S3), 33% (S4), 20% (S5), and 5% (S6). DESI images were acquired at a spatial resolution of 400 µm per pixel.

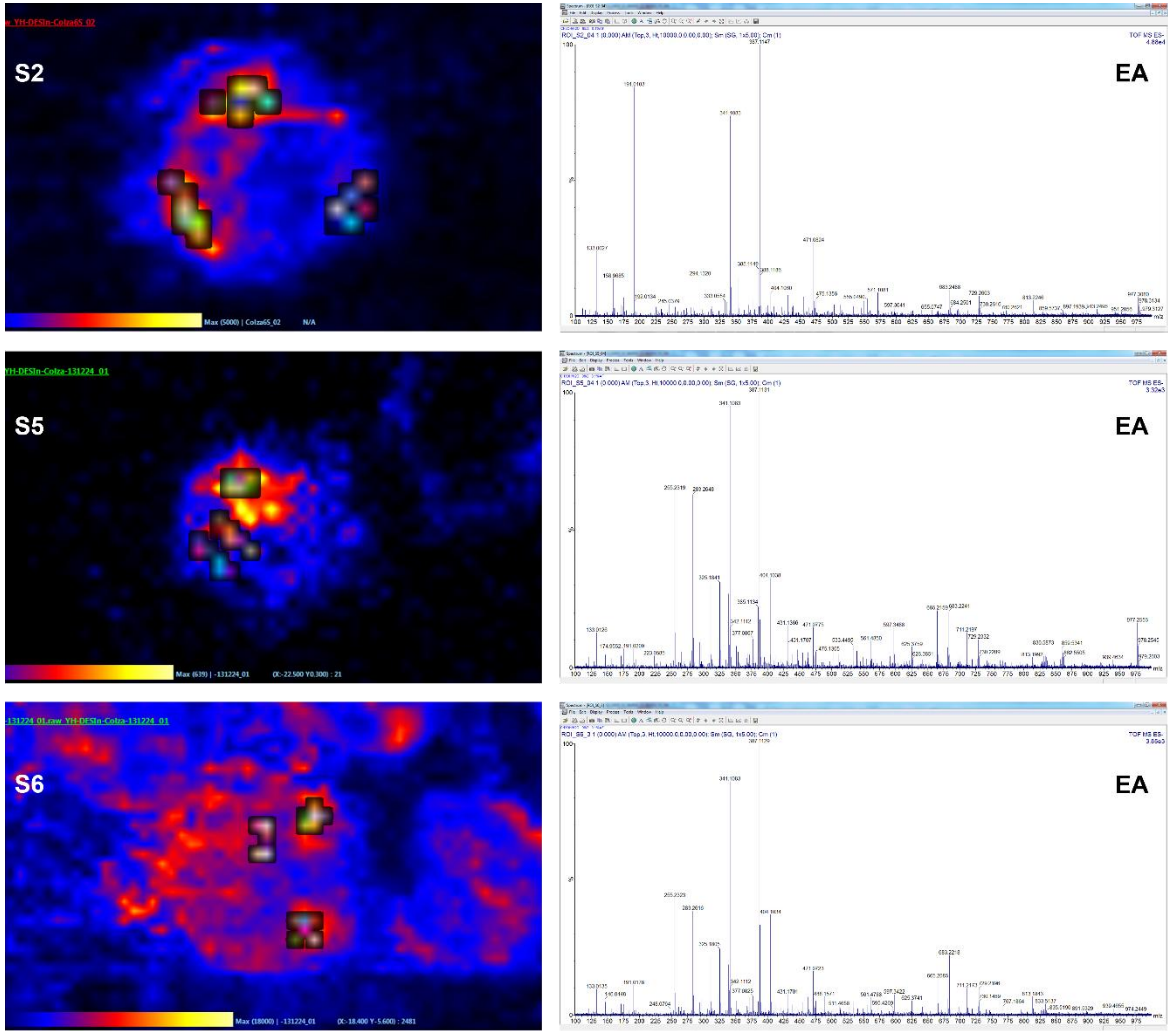

Fig. S2. Representative DESI pixels (left) from each *Brassica napus* L. seed compartment at three developmental stages defined by seed water content (% fresh weight): 52% (S2), 20% (S5), and 5% (S6). Seed compartments include the embryonic axis (EA), inner cotyledons (IC), and outer cotyledons (OC). The corresponding mean mass spectrum from the embryonic axis at each developmental stage is shown on the right.

	S1	S2	S3	S4	S5	S6
**Sucrose**	7.15	6.91	8.94	9.95	12.98	4.40
**Malate**	0.92	2.18	0.24	0.67	0.25	0.53
**Citrate**	2.41	0.69	0.16	0.54	0.12	0.82
**Glucose**	3.24	0.26	0.07	0.08	0.07	0.01
**Fructose**	3.97	0.15	0.21	0.15	0.12	0.06
**Myo-inositol**	0.45	0.24	0.06	0.78	0.07	0.03
**Galactose**	0.31	0.03	0.07	0.05	0.03	0.07
**Galactinol**	0.00	0.05	0.10	0.10	0.08	0.15
**Raffinose**	0.00	0.06	0.16	0.09	0.09	0.03
**Succinate**	0.35	0.12	0.06	0.07	0.10	0.07
**Glycerate**	0.33	0.01	0.07	0.04	0.03	0.01
**Fumarate**	0.22	0.22	0.15	0.14	0.11	0.07
**Histidine**	0.05	0.00	0.00	0.00	0.00	3.14
**Asparagine**	0.19	0.22	0.66	0.61	0.91	2.53
**Serine**	1.32	0.13	0.08	0.29	0.09	0.20
**Glutamine**	2.57	0.59	0.42	0.31	0.25	0.22
**Glycine**	0.29	0.38	0.20	0.19	0.36	0.12
**Aspartate**	0.17	0.14	0.19	0.12	0.22	1.66
**Glutamate**	0.37	0.29	0.16	0.60	0.47	3.57
**Threonine**	0.17	0.08	0.20	0.19	0.21	0.17
**Alpha-Alanine**	0.48	0.26	0.41	0.09	0.37	0.10
**Gaba**	2.84	0.35	0.15	0.40	0.24	0.13
**Proline**	0.25	0.22	0.14	0.02	0.05	0.28
**Lysine**	0.27	0.13	0.24	0.14	0.15	0.25
**Tyrosine**	0.17	0.15	0.26	0.08	0.11	0.26
**Valine**	0.27	0.08	0.17	0.03	0.12	0.07
**Isoleucine**	0.16	0.11	0.09	0.16	0.00	0.00
**Leucine**	0.14	1.96	0.28	0.36	0.52	1.38
**Phenylalanine**	0.21	0.33	0.14	0.03	0.07	0.03

Table S1. Standard deviations (n = 3) associated with the contents of major sugars, polyols, amino acids, and organic acids (µmol·g^{-1} dry weight) in *Brassica napus* L. seeds across six developmental stages. Stages were defined by seed water content (% fresh weight): 75% (S1), 52% (S2), 39% (S3), 33% (S4), 20% (S5), and 5% (S6).

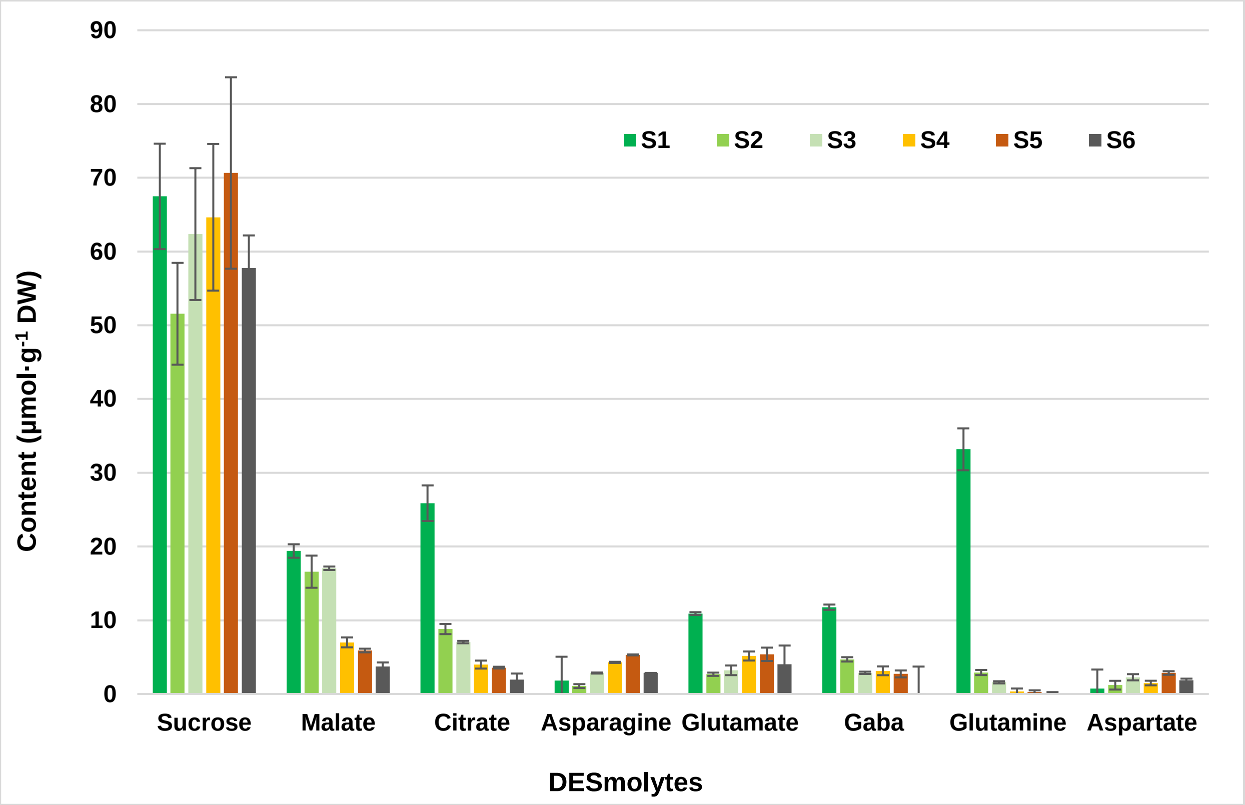

Fig. S3. Contents of selected DESmolytes, expressed in µmol/g of dry weight (µmol·g^{-1} DW), in *Brassica napus* L. seeds across six developmental stages. Stages were defined by seed water content expressed as a percentage of fresh weight (% FW): 75% (S1), 52% (S2), 39% (S3), 33% (S4), 20% (S5), and 5% (S6). Values represent means ± standard deviation (n = 3).

Analyzed solution	Adduct m/z (ESI$^-$)	Intensity	Fragments (10 eV)	Constituents
**Sucrose, dipotassium L-malate, tripotassium citrate, gaba, L-asparagine, L-glutamine, potassium L-aspartate and potassium L-glutamate**	444.1704	6 x10^3	102.0563 + 341.1076	Sucrose + Gaba
	473.1604	0.5 x10^4	131.0455 + 341.1076	Sucrose + Asparagine
	474.1468	0.5 x10^4	132.0298 + 341.1076	Sucrose + Aspartate
	475.1293	1.5 x10^4	133.0140 + 341.1095	Sucrose + malate
	487.1751	0.75 x10^5	145.0610 + 341.1072	Sucrose + Glutamine
	488.1604	0.75 x10^4	146.0454 + 341.1074	Sucrose + Glutamate

Table S2. Detected heterodimers in an artificial solution containing putative DESmolytes identified in *Brassica napus* L. seeds. The solution was analyzed by direct infusion using electrospray source (ESI$^-$). Exact masses, intensities and constituents of the detected sucrose-based heterodimers, as well as their fragments are indicated.

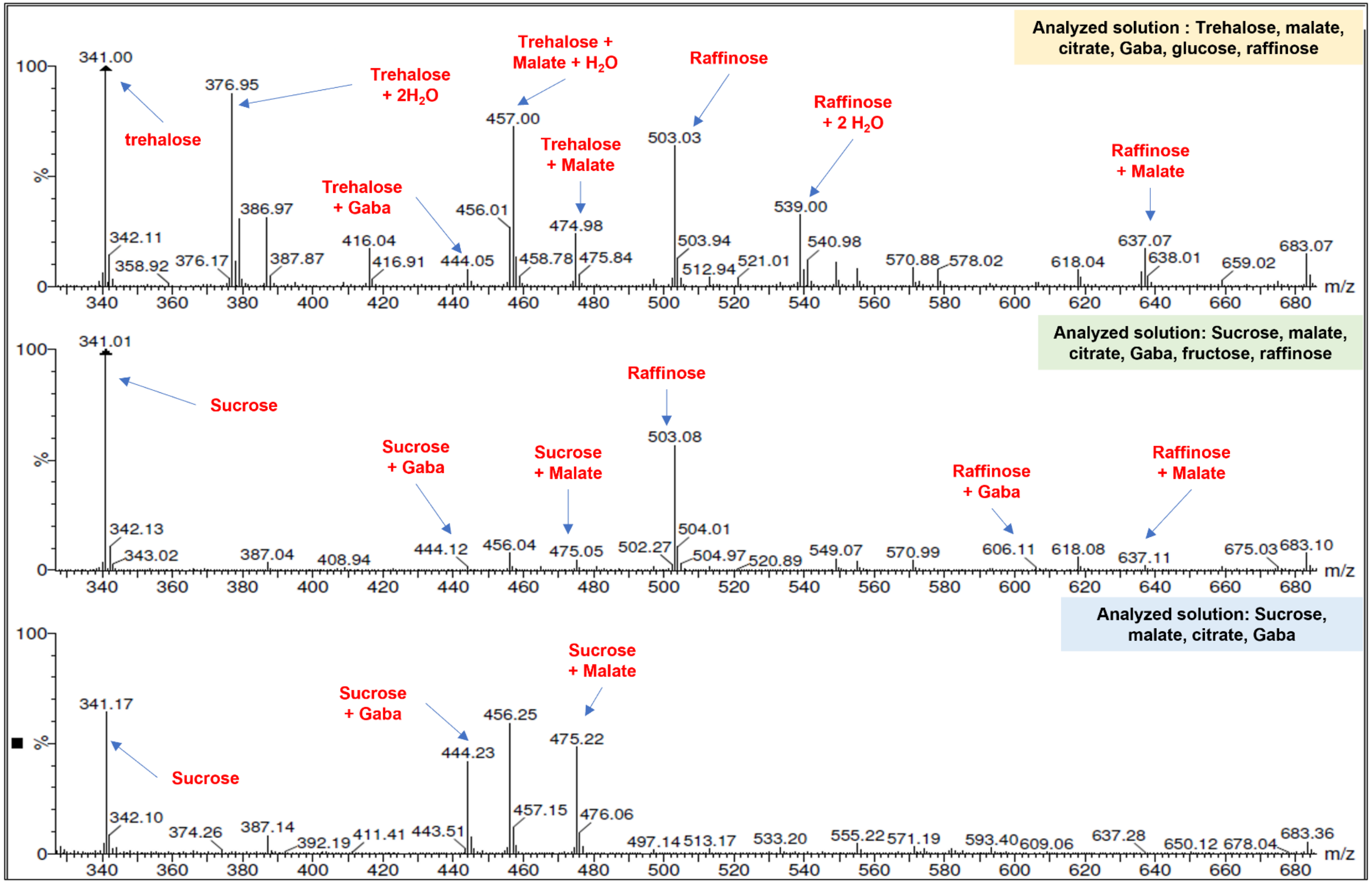

Fig. S4. Mass spectra representing a comparative analysis of three standard solutions containing DESmolytes at equimolar concentration. The 3 solutions contain the same DESmolytes, except for the sugars, which are different in each solution. Adducts formed between DESmolytes are identified in red and indicated with a blue arrow.

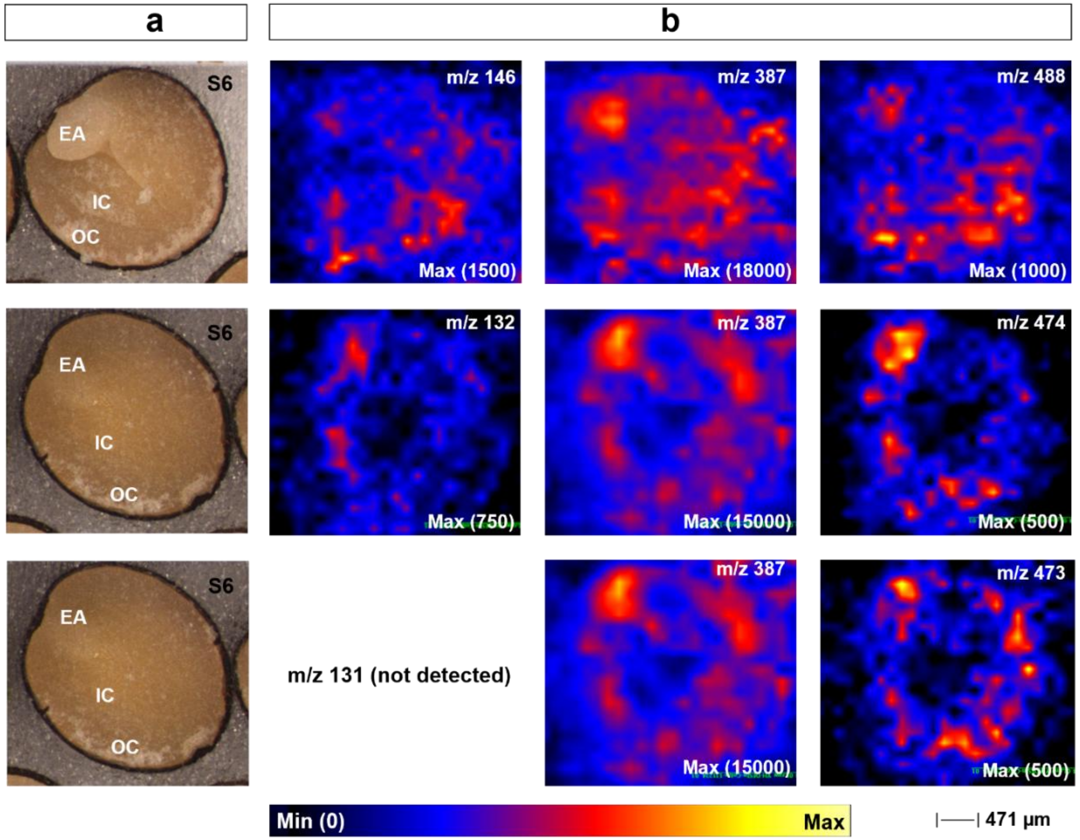

Fig. S5. Spatial distribution of additional DESmolytes and their heterodimers in dry *Brassica napus* L. seeds. (**a**): Microscopic images showing seed compartments in two representative seeds with 5% water content (relative to fresh weight): embryonic axis (EA), inner cotyledons (IC), and outer cotyledons (OC). (**b**): DESI images illustrating the spatial distribution of glutamate (m/z 146), sucrose (m/z 387), and aspartate (m/z 132), as well as heterodimers formed between glutamate and sucrose (m/z 488), aspartate and sucrose (m/z 474), and sucrose and asparagine (m/z 473) within seed tissues. Signal intensity ranges from minimum (black, 0) to maximum (yellow), with the maximum intensity for each molecule indicated in parentheses. DESI images were acquired at a spatial resolution of 100 µm per pixel.

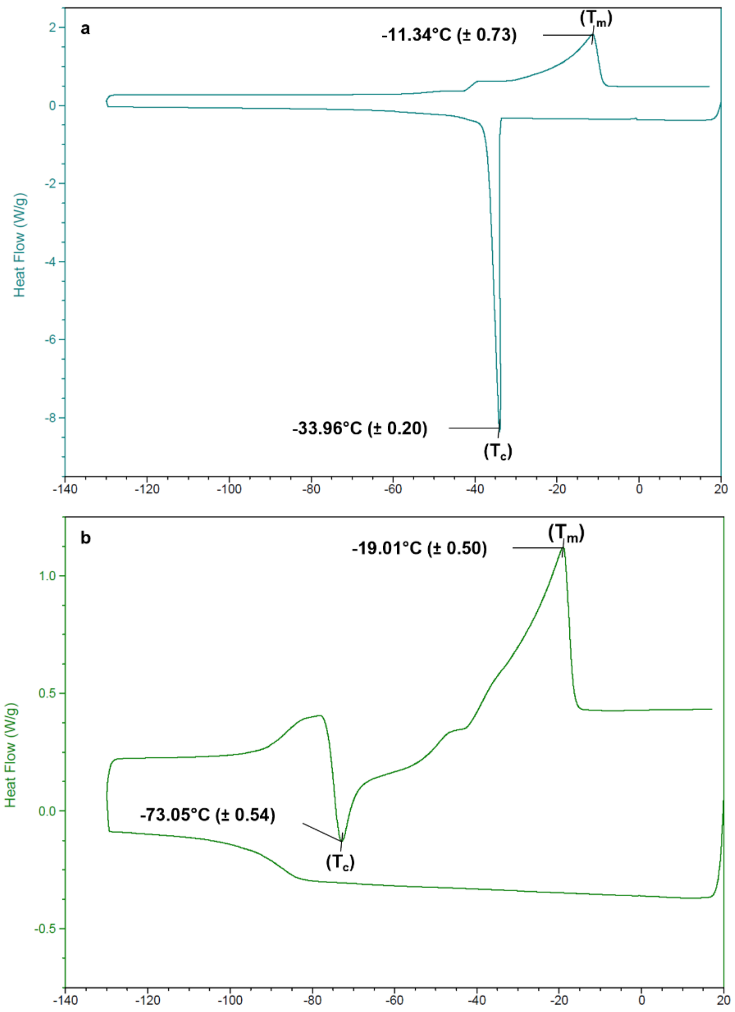

Fig. S6. Phase transitions of sucrose/dipotassium malate (1/1; mol/mol) mixtures at different water contents. Panels (a) and (b) represent the full thermograms, including both cooling and heating cycles, at 50% and 40% water content (w/w) respectively. Analyses were carried out between 20°C and -130°C with a heat flow of 10°C·min^{-1}. Tm: melting temperature; Tc: crystallization temperature.

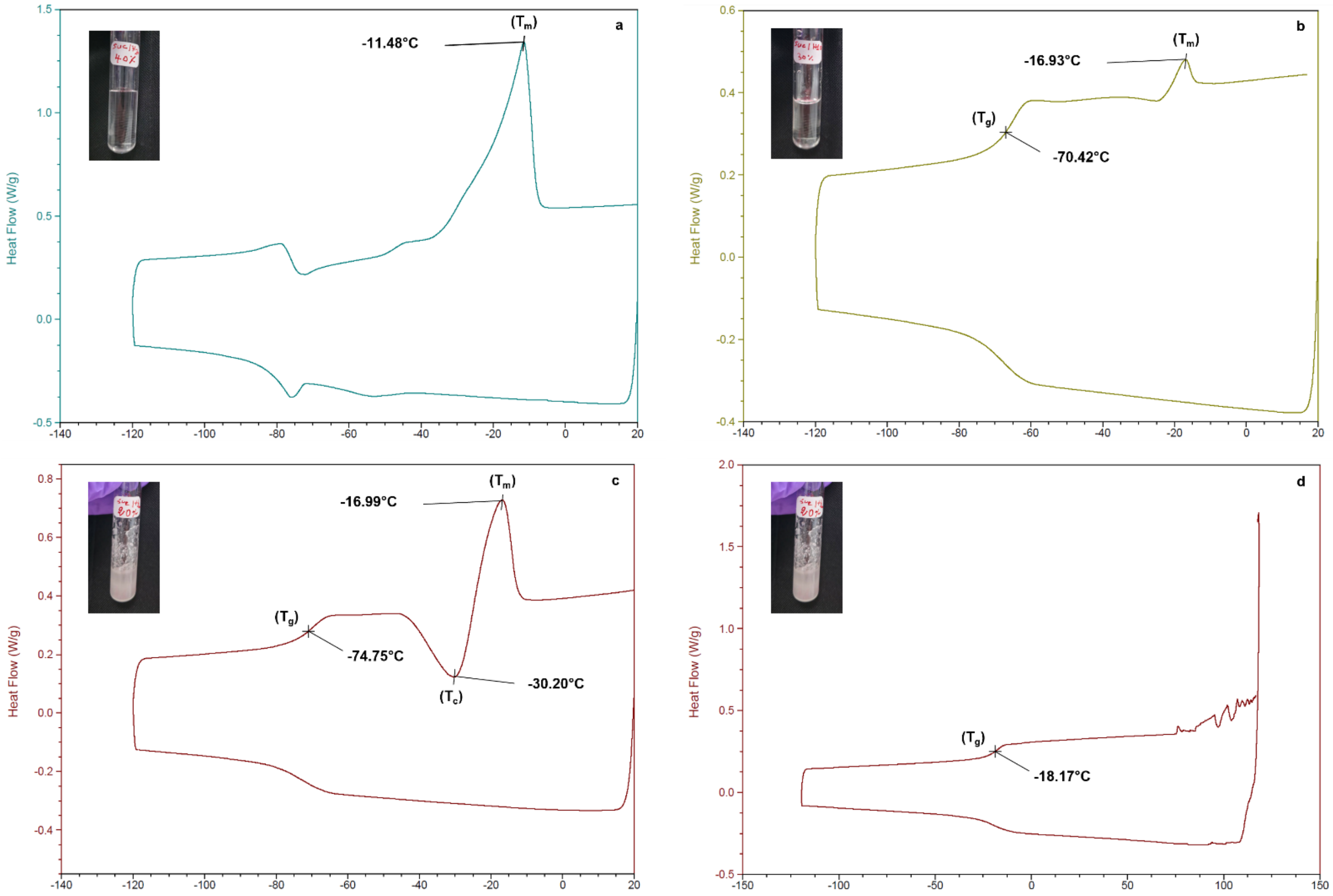

Fig. S7. Phase transitions of sucrose/water solution at different water contents. Panels (a-c) represent thermograms of the sucrose solution at 40%, 30%, and 20% water content (w/w). The thermograms were obtained between 20°C and -130°C. Panel (d) represents a thermogram of the solution at 20% water content (w/w) obtained between 120°C and -130°C. Chemical degradation, detected after heating above 100°C, is illustrated by multiples small peaks in panel (d). Heat flow was 10°C·min^{-1}. Tm: melting temperature; Tc: crystallization temperature; Tg: glass transition temperature.

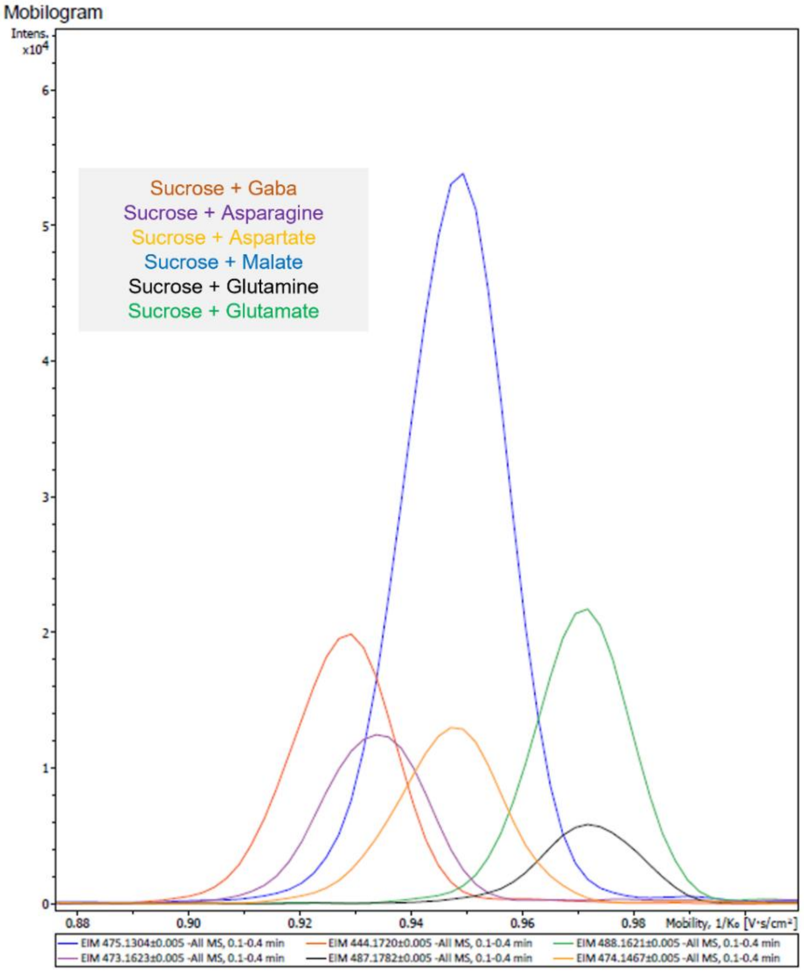

Fig. S8. Ion mobility of detected heterodimers in *Brassica napus* L. seed extract at the S3 developmental stage (39% water content, % fresh weight). Each peak in the mobilogram representing the ion mobility of each dimer indicated with the same color.
